# The *De Novo* Cytosine Methyltransferase DRM2 Requires Intact UBA Domains and a Catalytically Mutated Paralog DRM3 during RNA–Directed DNA Methylation in *Arabidopsis thaliana*

**DOI:** 10.1371/journal.pgen.1001182

**Published:** 2010-10-28

**Authors:** Ian R. Henderson, Angelique Deleris, William Wong, Xuehua Zhong, Hang Gyeong Chin, Gregory A. Horwitz, Krystyna A. Kelly, Sriharsa Pradhan, Steven E. Jacobsen

**Affiliations:** 1 Department of Molecular, Cell, and Developmental Biology, University of California Los Angeles, Los Angeles, California, United States of America; 2 Department of Plant Sciences, University of Cambridge, Cambridge, United Kingdom; 3 New England Biolabs, Ipswich, Massachusetts, United States of America; 4 Howard Hughes Medical Institute, University of California Los Angeles, Los Angeles, California, United States of America; The Salk Institute for Biological Studies, United States of America

## Abstract

Eukaryotic DNA cytosine methylation can be used to transcriptionally silence repetitive sequences, including transposons and retroviruses. This silencing is stable between cell generations as cytosine methylation is maintained epigenetically through DNA replication. The *Arabidopsis thaliana* Dnmt3 cytosine methyltransferase ortholog DOMAINS REARRANGED METHYLTRANSFERASE2 (DRM2) is required for establishment of small interfering RNA (siRNA) directed DNA methylation. In mammals PIWI proteins and piRNA act in a convergently evolved RNA–directed DNA methylation system that is required to repress transposon expression in the germ line. *De novo* methylation may also be independent of RNA interference and small RNAs, as in *Neurospora crassa*. Here we identify a clade of catalytically mutated DRM2 paralogs in flowering plant genomes, which in *A.thaliana* we term DOMAINS REARRANGED METHYLTRANSFERASE3 (DRM3). Despite being catalytically mutated, *DRM3* is required for normal maintenance of non-CG DNA methylation, establishment of RNA–directed DNA methylation triggered by repeat sequences and accumulation of repeat-associated small RNAs. Although the mammalian catalytically inactive Dnmt3L paralogs act in an analogous manner, phylogenetic analysis indicates that the DRM and Dnmt3 protein families diverged independently in plants and animals. We also show by site-directed mutagenesis that both the DRM2 N-terminal UBA domains and C-terminal methyltransferase domain are required for normal RNA–directed DNA methylation, supporting an essential targeting function for the UBA domains. These results suggest that plant and mammalian RNA–directed DNA methylation systems consist of a combination of ancestral and convergent features.

## Author Summary

Nuclear DNA quantity varies widely between species and is poorly correlated with gene number. Variation in genome size can be explained by differing amounts of repetitive DNA. Repetitive DNA may be mobile, meaning it can increase its copy number within genomes. To prevent this, plants and animals suppress expression of repeats, often by marking the repeated sequence with a methyl group on cytosine bases. DRM2 is an enzyme capable of establishing this methylation, which can be guided to target sequences by short complementary RNA guides. As repeated sequences are prone to generate short RNAs they are efficiently recognized and silenced. We show that DRM2 requires a related inactive DRM3 protein to normally silence repeated sequences. A similar situation exists in mammals, where active and inactive DNA methyltransferases act together to silence repeats. We also demonstrate that non-catalytic regions of the DRM2 enzyme are functionally important, which we speculate to be involved in targeting the enzyme to the genome. Although plant and mammal RNA–directed DNA methylation systems share key similarities, there are important mechanistic differences, meaning that they are likely to have arisen convergently.

## Introduction

Repetitive DNA in eukaryotic genomes is often transcriptionally silent, due to genome defense mechanisms directed against transposons and other mobile DNA [1], [2]. In plant genomes repetitive sequences are associated with DNA cytosine methylation [3]–[8], which is required for transcriptional silencing and suppression of transposon activity [9]–[12]. As DNA methylation can be maintained epigenetically through DNA replication, this mark serves to stably repress expression of repeated sequences following cell division [2], [13]. Gene promoters in *A.thaliana* are generally not DNA methylated, though gene bodies (open reading frames) may contain DNA methylation in the CG sequence context [3]–[8]. Eukaryotic DNA methylation is catalyzed by cytosine methyltransferases that share ancestry with prokaryotic restriction modification enzymes and are characterized by 10 conserved catalytic motifs [13], [14]. During catalysis a methyl group is transferred from the donor molecule S-adenosyl methionine to the carbon-5 position of the cytosine base, using an essential cysteine residue in catalytic motif IV [13], [14].

Three functional classes of DNA methyltransferase exist in *A.thaliana*; *METHYLTRANSFERASE1* (*MET1*) (orthologous to mammalian *Dnmt1*) which maintains CG methylation, *CHROMOMETHYLASE3* (*CMT3*) (plant specific) which maintains methylation in non-CG sequence contexts and *DOMAINS REARRANGED METHYLTRANSFERASE2* (*DRM2*) (orthologous to *Dnmt3a*/*Dnmt3b*) which both *de novo* methylates DNA and maintains non-CG methylation redundantly with *CMT3* [2]. The *drm2* mutation blocks all *de novo* DNA methylation driven by repeat containing transgenes [15]–[17]. This phenotype is shared with mutations in a diverse set of RNA interference and chromatin proteins including *NUCLEAR RNA POLYMERASE D1* (*NRPD1*), *NUCLEAR RNA POLYMERASE E1* (*NRPE1*), *RNA DEPENDENT RNA POLYMERASE2* (*RDR2*), *DICER-LIKE3* (*DCL3*), *ARGONAUTE4* (*AGO4*), *DEFECTIVE IN RNA DIRECTED DNA METHYLATION 1* (*DRD1*), *DEFECTIVE IN MERISTEM SILENCING1* (*DMS1*), *INVOLVED IN DE NOVO 2* (*IND2*), *SUPPRESSOR OF VARIEGATION 3-9 HOMOLOGUE 2* (*SUVH2*) and *SUPPRESSOR OF VARIEGATION 3-9 HOMOLOGUE 9* (*SUVH9*) [16]–[34]. These genes act in a pathway that generates short interfering RNAs (siRNAs) which can guide DRM2 DNA methyltransferase activity to homologous genomic sequences [2]. This is reflected in the strong correlation between genomic repeats, DNA methylation and siRNAs in *A.thaliana* [3]–[5], [7], [8]. Small RNAs are also required for *DRM2* to maintain non-CG DNA methylation and silencing at a subset of endogenous loci, though this function is partially redundant with a second pathway consisting of *CMT3* and the histone H3K9 methyltransferase *KRYPTONITE/SUPPRESSOR OF VARIEGATION HOMOLOGUE4* [17], [35]–[37].

The mammalian DRM2 orthologs, Dnmt3a and Dnmt3b, are required to *de novo* methylate integrated retroviral sequences and imprinted genes [38], [39]. Dnmt3a forms a complex with a non-catalytic paralog Dnmt3L, which is also required for normal patterns of DNA methylation *in vivo* [40]–[46]. The C-terminal domains of Dnmt3a and Dnmt3L have been co-crystallized and shown to form a hetero-tetrameric complex, which promotes Dnmt3a methyltransferase activity [42]–[46]. The N-terminal PHD domain of Dnmt3L interacts with unmethylated H3K4 histone tails and recruits Dnmt3a to specific loci, including imprinted genes [45], [46]. Thus *de novo* methylation mediated by Dnmt3a requires interaction with non-catalytic Dnmt3L, which both stimulates catalysis and recruits the complex to chromatin. Animal PIWI proteins bind germ cell specific 25–30 nucleotide piRNAs, many of which are homologous to repeated sequences [47]. The mouse PIWI mutants *miwi*, *mili* and *miwi2* are sterile and have defects in transposon *de novo* methylation in male germ cells, with a phenotype very similar to *dnmt3l* [40], [48]–[52]. Hence, small RNAs are required to target a subset of *de novo* DNA methylation in mammals, though as plants lack PIWI domains and piRNA, these systems appear to have evolved independently. *De novo* DNA methylation can also be independent of small RNAs and RNA interference, as in *Neurospora crassa* [53].

Here we describe a novel protein required for RNA–directed DNA methylation in *A.thaliana*, DOMAINS REARRANGED METHYLTRANSFERASE3 (DRM3). Based on sequence analysis and genetic observations DRM3 appears to be catalytically mutated DNA methyltransferase paralog. A conserved clade of *DRM3* genes is found throughout angiosperm genomes. Although *DRM3* cannot compensate for loss of *DRM2* function *in vivo*, it is required for normal establishment and maintenance of RNA–directed DNA methylation and accumulation of specific repeat-associated siRNA. This is analogous to the function of Dnmt3L in mammals, though phylogenetic analysis indicates that DRM3 and Dnmt3L proteins diverged independently in plant and animal lineages. We also demonstrate using site-directed mutagenesis that both the catalytic methyltransferase domain and UBA domains of DRM2 are required for RNA–directed DNA methylation. We speculate that the UBA domains are involved in targeting DRM2 activity during *de novo* DNA methylation, potentially by recognizing a specific chromatin state. These findings extend parallels between plant and mammalian RNA–directed DNA methylation systems, despite their independent evolution.

## Results

### A conserved clade of catalytically mutated DRM3 paralogs within angiosperms

**Figure 1:**
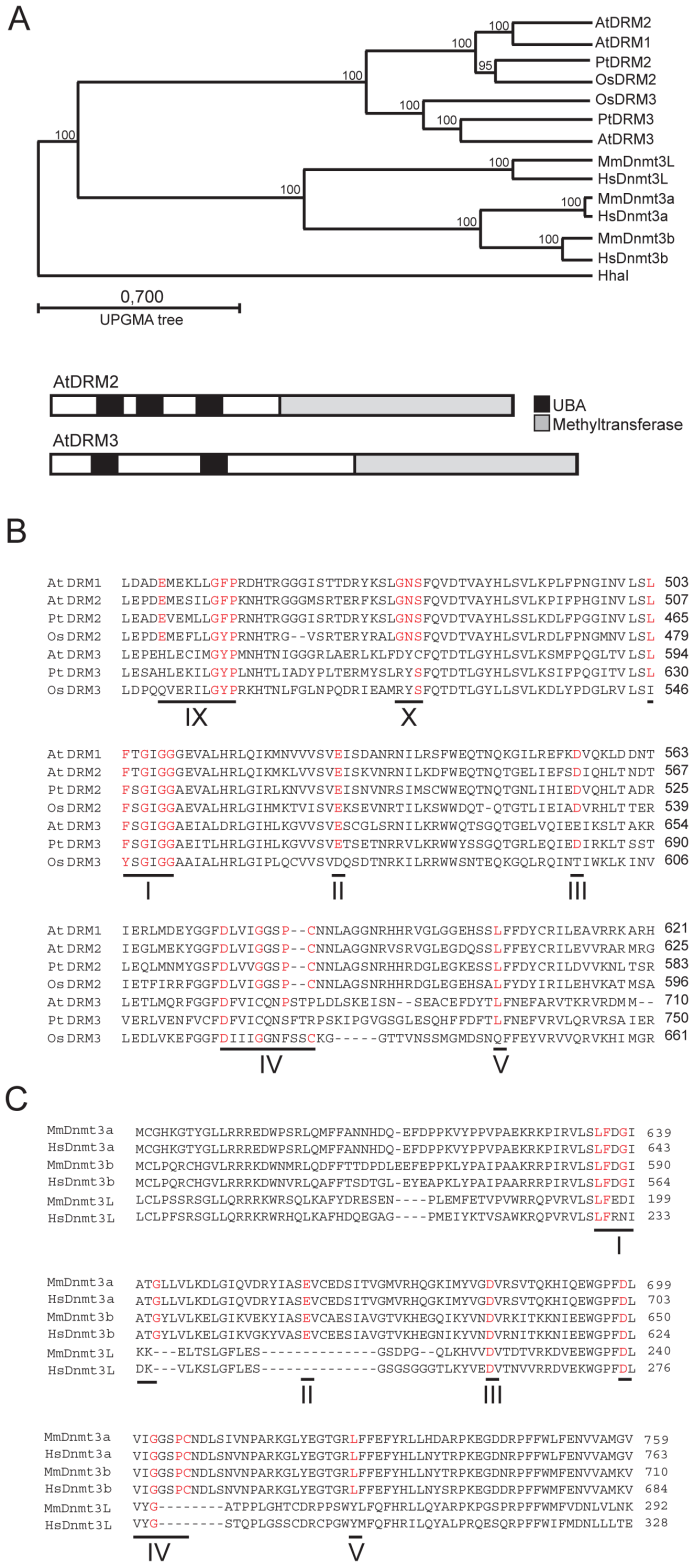
A conserved clade of catalytically mutated DRM cytosine methyltransferase paralogs in angiosperms. (A) Phylogenetic relationships between DRM and Dnmt3 proteins (tree constructed using Unweighted Pair Group Method with Arithmetic Means (UPGMA)). Beneath are graphical representations of DRM2 and DRM3 (UBA domains are shaded black and cytosine methyltransferase domains are shaded grey). (B) Sequence alignment of DRM methyltransferase domains. Conserved catalytic residues are highlighted in red and numbered according to established nomenclature. (C) Sequence alignment of Dnmt3 methyltransferase domains, labeled as in (B). NCBI accession numbers or gene numbers for the proteins analyzed are (*Arabidopsis thaliana*) AtDRM1 At5g15380, AtDRM2 At5g14620, AtDRM3 At3g17310, (*Populus trichocarpa*) PtDRM2 DMT905, PtDRM3 DMT907, (*Oryzae sativa*) OsDRM2 Os3g02010, OsDRM3 Os5g04330, (*Homo sapiens*) HsDnmt3a NP_783328, HsDnmt3b CAB53071, HsDnm3L BAA95556, (*Mus musculus*) MmDnmt3a O88508, MmDnmt3b CAM27225, MmDnmt3L AAH83147 and (*Haemophilus parahaemolyticus*) HhaI P05102.

The *A.thaliana* genome encodes a previously uncharacterized protein with high amino acid identity to DRM2, which we term DRM3 (At3g17310) (Figure 1A and 1B). Like DRM2, DRM3 contains N-terminal ubiquitin associated (UBA) domains and a C-terminal region which shares identity with the Dnmt3 cytosine methyltransferase domain, but whose catalytic motifs are rearranged such that motifs VI–X precede I–V (Figure 1A and 1B) [54]. During catalysis cytosine methyltransferases form a covalent bond between a conserved cysteine in motif IV and carbon-6 of the cytosine base [14]. The catalytic cysteine is preceded by an invariant proline, which hydrogen bonds to exo-cyclic NH_2_ groups of cytosine to promote specific recognition and stabilize interaction between the base and catalytic site [14]. Close inspection of the DRM3 methyltransferase domain reveals the absence of highly conserved residues and notably the invariant proline-cysteine sequence is absent from motif IV (Figure 1B). Additionally, DRM3 lacks a conserved glutamic acid within motif IX and glycine within motif X (Figure 1B). It is likely that the absence of these residues inactivate DRM3 cytosine methyltransferase activity, because a *drm2* mutation alone is sufficient to block *de novo* DNA methylation [15]–[17].

BLAST searches of the angiosperm *Populus trichocarpa* and *Oryzae sativa* genomes reveals a clade of DRM3 proteins shared between these species, indicating that distinct DRM2 and DRM3 proteins are conserved and were present prior to the divergence of the monocots and dicots (Figure 1A). All members of the DRM3 clade lack conserved motif IV proline-cysteine, motif IX glutamic acid and motif X glycine residues (Figure 1B). Although OsDRM3 possesses a cysteine within motif IV, it lacks a preceding proline and therefore is predicted to be catalytically inactive (Figure 1B) [14]. The mammalian Dnmt3L proteins also carry mutations in key catalytic motifs, including the catalytic proline-cysteine sequence in motif IV [36]–[39] (Figure 1C). However, phylogenetic analysis indicates that the Dnmt3 and DRM protein families diverged independently in plants and mammals (Figure 1A). This scenario is supported by the fact that all DRM proteins share an identical rearrangement of their catalytic motifs (Figure 1B) [54]. As Dnmt3L proteins are important for mammalian *de novo* DNA methylation we sought to test whether *DRM3* is analogously required for RNA–directed DNA methylation in *A.thaliana*.

### *DRM3* is required for maintenance of non-CG DNA methylation

**Figure 2:**
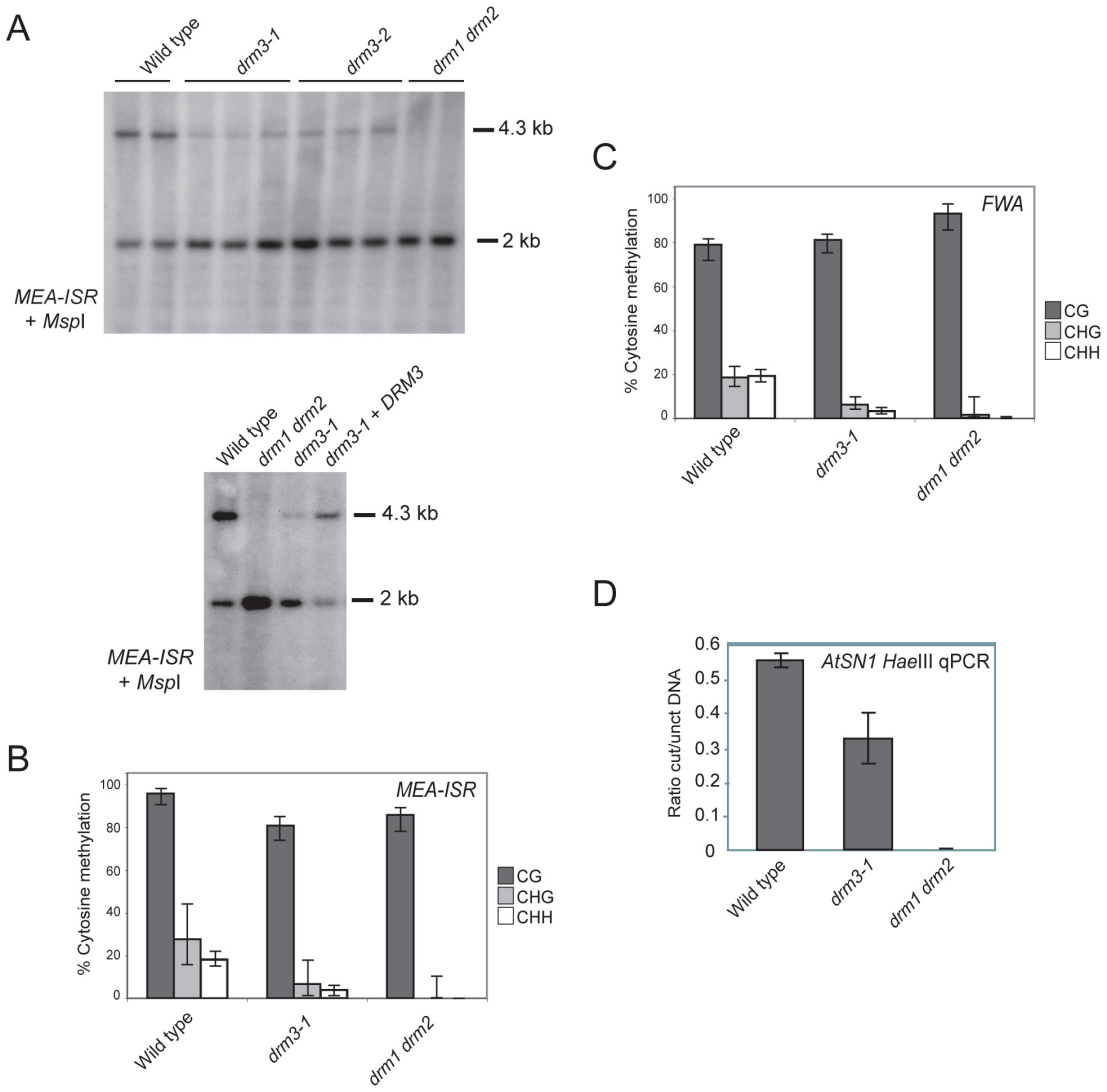
*DRM3* is required for normal maintenance of non-CG methylation. (A) Southern blots hybridized for the *MEA-ISR* repeat using DNA digested with the methyl-sensitive restriction enzyme *Msp*I. The 4.3 kb band and 2 kb bands represent digested and undigested DNA respectively. (B) Graphical representation of bisulfite sequencing at *MEA-ISR* with % cytosine methylation shown for each genotype. CG sequence contexts are shaded black, CHG are shaded grey and white represents CHH. The 95% confidence limits shown are given by the Wilson score interval. (C) Graphical representation of bisulfite sequencing at the methylated promoter repeats of the *FWA* endogene with % cytosine methylation shown for each genotype and shaded according to sequence context as in (B). (D) Quantitative PCR measurement of *AtSN1* genomic DNA following restriction endonuclease digestion with DNA methylation sensitive enzyme *Hae*III. The value on the *y*-axis represents the ratio of DNA measured by quantitative PCR with and without *Hae*III digestion. The data represents three biological replicates and the error bars represent standard error.

To test whether *DRM3* is required for normal DRM2 function we obtained two independent transfer-DNA (T-DNA) insertions in this gene (*drm3-1* and *drm3-2*) (Figure S1). We tested whether the *drm3* mutations have a defect in maintenance of non-CG methylation at the *MEA-ISR* endogenous tandem repeat [36]. Non-CG methylation at *MEA-ISR* is lost in *drm2* and RNAi mutants such as *rdr2*, which can be assayed by Southern blotting and hybridization after genomic DNA digestion with the methyl-sensitive restriction endonuclease *Msp*I [36]. The higher molecular weight 4.3 kb band hybridizing to *MEA-ISR* represents inhibition of *Msp*I digestion by cytosine methylation at a single CHG site [36]. The lower 2 kb band represents digested DNA and in wild type is present at levels comparable to the upper band (Figure 2A) [36]. In *drm1 drm2* mutants DNA methylation at this site is eliminated and the 4.3 kb band on the Southern blot is absent (Figure 2A) [36]. Using this assay we observed that both *drm3* alleles show a reduction of methylation at *MEA-ISR* to a level intermediate between wild type and *drm1 drm2* (Figure 2A). This methylation phenotype could be complemented by transformation of *drm3-1* with a genomic fragment containing the *DRM3* gene (Figure 2A). As the Southern blot assay measures methylation at a single CHG site we also sequenced the *MEA-ISR* locus after bisulfite conversion. This independently corroborated that *drm3-1* shows an intermediate level of non-CG methylation at *MEA-ISR* between that of wild type and *drm1 drm2* (Figure 2B, Table S1 and Figure S3). Although significant differences in CG methylation are observed between genotypes, individual CG sites remain highly methylated in all samples (>70%) and reductions in the percentage of methylated sites are less than for non-CG methylation (*drm3-1* shows a 16% reduction in CG versus 76% reduction in both types of non-CG methylation, while *drm1 drm2* shows an 11% reduction in CG versus reduction to absence of non-CG methylation) (Figure 2B, Table S1 and Figure S3). Furthermore, *drm1 drm2* and *drm3* do not share developmental phenotypes characteristic of mutants that fail to maintain CG methylation such as *met1*, *ddm1* and *vim1 vim2 vim3* [55]–[57].

To further characterize the *drm3* phenotype we performed sodium bisulfite sequencing across the promoter tandem repeats of the *FWA* endogene. These repeats are cytosine methylated in wild type and show an elimination of non-CG methylation in *drm1 drm2* [36]. As for *MEA-ISR*, we observed that *drm3-1* shows a strong reduction of non-CG methylation at *FWA* to a level intermediate between wild type and *drm1 drm2* (Figure 2C, Table S2 and Figure S3). We also analyzed maintenance of DNA methylation at the SINE retroelement *AtSN1* using *Hae*III restriction endonuclease digestion of genomic DNA followed by quantitative PCR [18], [27]. *Hae*III digestion is DNA methylation sensitive, meaning that methylated sequences are protected from digestion. In wild type *AtSN1* is densely methylated and resistant to *Hae*III cleavage, whereas in *drm1 drm2* methylation is greatly reduced and *AtSN1* DNA is digested and as a consequence not amplified (Figure 2D). We repeated this assay in *drm3-1* and again observed an intermediate phenotype between wild type and *drm1 drm2*, indicating a defect in maintenance of DNA methylation at *AtSN1* (Figure 2D). We conclude from these observations that DRM3 is required for normal maintenance of non-CG DNA methylation *in vivo*. However, as non-CG methylation is not completely eliminated in *drm3*, it is not absolutely required for DRM2 activity.

### *DRM2* mRNA and protein expression are independent of *DRM3*

One explanation for *drm3* phenotypes could be an influence on *DRM2* expression or protein stability. To test this idea we first measured *DRM2* mRNA levels using quantitative reverse-transcriptase PCR. This demonstrated that *DRM2* mRNA accumulated normally in *drm3* mutants relative to wild type (Figure S1). To test the accumulation of DRM2 protein we crossed a complementing *DRM2-Myc* transgene into *drm3-1* and performed western blotting using α-Myc antibodies [58]. DRM2-Myc protein accumulated normally in *drm3-1* relative to wild type (Figure S1). We conclude from these experiments that DRM2 expression is independent of *DRM3*.

### *DRM3* is required for normal establishment of RNA–directed DNA methylation

**Figure 3:**
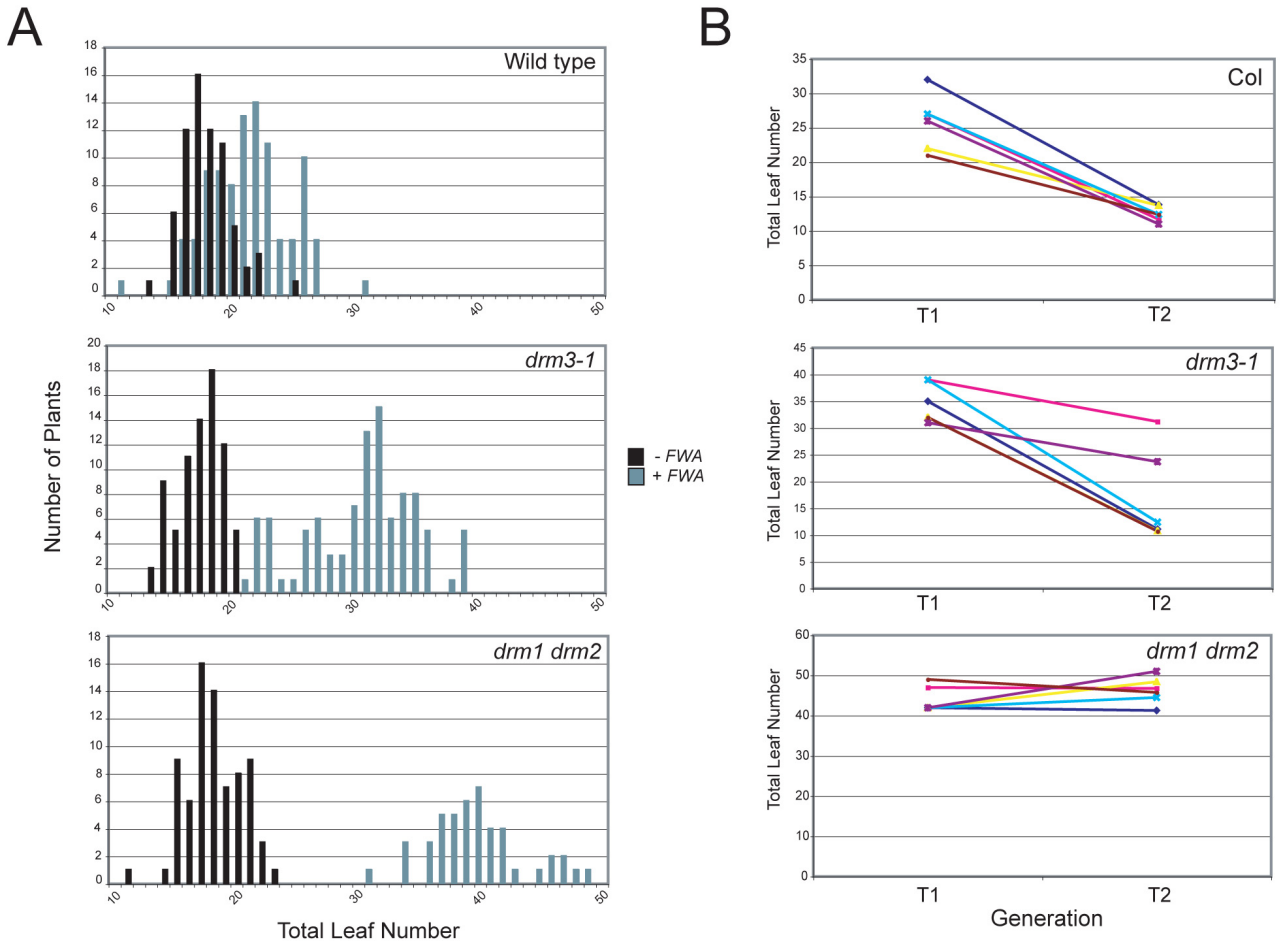
*DRM3* is required for normal establishment of RNA–directed DNA methylation. (A) Histograms showing flowering-time distribution of untransformed plants (black bars) or T_1_ *FWA* transformant plants (grey bars). Flowering-time is measured as total leaf number and plotted along the *x*-axis, with number of plants on *y*-axis. (B) Graph showing flowering time (total leaf number) of individual T_1_ plants connected to the average flowering time of their T_2_ progeny. Each colour represents an individual T_1_–T_2_ family.

*DRM2* is required for both maintenance of non-CG methylation and *de novo* establishment of methylation in all sequence contexts [15], [35], [36]. To test whether *DRM3* is also required for normal establishment of RNA–directed DNA methylation we used *Agrobacterium*-mediated transformation with a tandem repeat containing *FWA* transgene [15], [16], [59]. In wild type endogenous *FWA* is transcriptionally silenced by DNA methylation at a set of promoter tandem repeats [59]. Loss of methylation at these repeats causes a dominant late-flowering phenotype, which can be quantified by counting leaf number before flowering [59]. When *FWA* is transformed into wild type *A.thaliana* the promoter repeats on the incoming transgene are efficiently methylated, causing transcriptional silencing [15], [16], [59]. However, *FWA* transformation into *drm2* or RdDM pathway mutants (e.g. *nrpd1*, *ago4*, *rdr2*, *dcl3*) leads to a failure in *de novo* methylation, meaning incoming *FWA* transgenes are expressed causing a dominant late-flowering phenotype in transformed individuals [15], [16]. Therefore, we transformed *FWA* into wild type (Col), *drm3-1* and *drm1 drm2* backgrounds and analyzed the flowering time of first generation transformed individuals (T_1_) as a measure of establishment of RNA–directed DNA methylation. Untransformed plants were grown alongside +*FWA* transformants as controls and analyzed for flowering time. As previously reported *FWA* transformants in wild type flowered slightly but significantly later than untransformed controls, reflecting incomplete establishment of silencing in the T_1_ generation (Figure 3A and Table 1) [15], [16]. In contrast, *drm1 drm2 FWA* transformants flowered far later due to a failure to silence the incoming transgenes (Figure 3A and Table 1) [15], [16]. We observed that *drm3-1 FWA* transformants showed a flowering time distribution intermediate between the wild type and *drm1 drm2* T_1_ populations (Figure 3A and Table 1). This parallels our observations for maintenance of non-CG methylation with *drm3-1* showing a defect in *DRM2-*mediated DNA methylation, though with a phenotype weaker than *drm1 drm2*.

**Table 1:** Average flowering-time of *FWA* T_1_ transformant populations.

	Total leaf number +/− standard error	
Genotype	Untransformed	+*FWA*
Col	17.8+/−0.25	21.5+/−0.83
*drm3-1*	17.0+/−0.21	30.5+/−0.82
*drm1 drm2*	18.0+/−0.24	41.1+/−0.70
*DRM2-Myc drm1 drm2*	15.39+/−0.18	27.8+/−0.39
*DRM2cat-Myc drm1 drm2*	17.06+/−0.24	52.86+/−1.13
*DRM2uba-Myc drm1 drm2*	18.09+/−0.18	51.51+/−1.06
Col	17.1+/−0.41	20.0+/−0.23
*drm3-1*	21.3+/−0.26	32.0+/−0.68
*dcl3-1*	19.1+/−0.25	36.2+/−0.53
*drm3-1 dcl3-1*	15.9+/−0.19	42.6+/−1.23
*drm1 drm2*	16.7+/−0.23	44.7+/−0.93

*FWA* silencing is frequently incomplete in the T_1_ generation and self-fertilization of transformed individuals results in an increase in *FWA* repeat DNA methylation and transcriptional silencing in the T_2_ progeny, which as a consequence flower earlier [59]. As *drm3* appears to reduce but not abolish *DRM2* activity, we predicted that wild type and *drm3-1* T_2_ populations would become earlier flowering relative to the T_1_, whereas *drm1 drm2* would not. Analysis of the flowering-time distribution of multiple T_2_ populations relative to their T_1_ parents showed this to be true (Figure 3B and Table S3). Consistent with a complete block to *de novo* DNA methylation in *drm1 drm2* T_2_ individuals did not flower significantly earlier, which they did in the case of wild type and *drm3-1* (Figure 3B and Table S3). Together, we interpret this as meaning that although *DRM2* is absolutely required for *de novo* DNA methylation *in vivo*, normal levels of methyltransferase activity also require non-catalytic *DRM3*.

Intermediate RNA–directed DNA methylation phenotypes between wild type and *drm1 drm2* have previously been observed in *dcl3* mutants [16], [60], [61]. DCL3 generates 24 nucleotide siRNAs with a specialized function in RNA–directed DNA methylation and transcriptional silencing, though it acts redundantly with the other DCLs in a locus-specific manner [16], [60]–[62]. We generated *drm3-1 dcl3-1* double mutants and tested for enhancement of defects during establishment of *FWA* RNA–directed DNA methylation, relative to the single mutants. We observed that *drm3-1 dcl3-1* double mutants showed a silencing defect comparable to *drm1 drm2*, which was greater than either *drm3-1* or *dcl3-1* alone (Table 1). We interpret this enhancement as meaning that *DRM3* and *DCL3* act genetically in parallel to promote DRM2 activity *in vivo*.

### Mutagenesis of DRM2 reveals a functional requirement for cytosine methyltransferase and UBA domains

**Figure 4:**
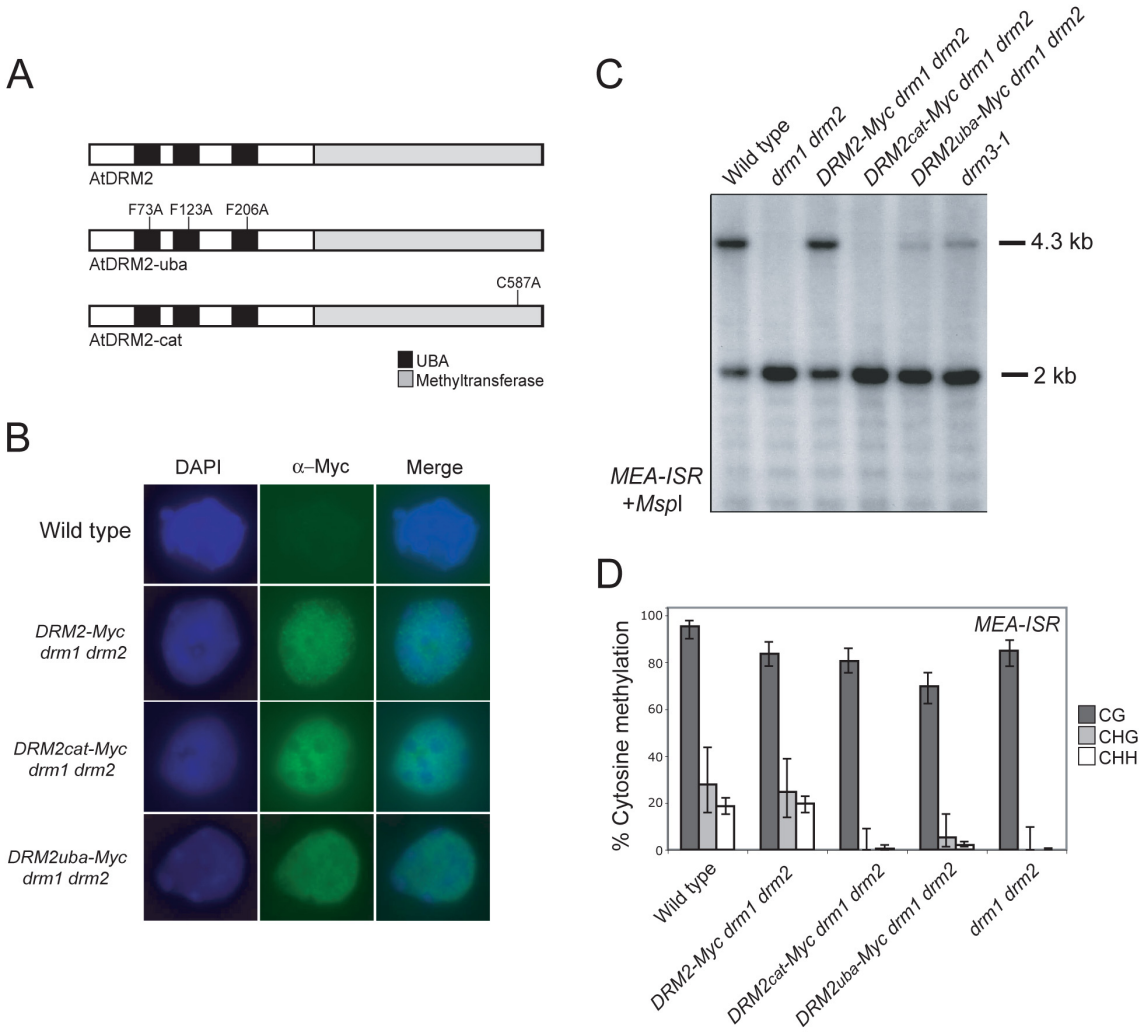
DRM2 catalytic and UBA domains are required for maintenance of non-CG methylation. (A) A Myc-epitope tagged *DRM2* transgene was mutated to introduce amino acid substitutions in the UBA domains or the methyltransferase domain, depicted graphically. (B) Micrographs of intact nuclei stained with DAPI and immunostained using α-Myc antibodies. (C) Southern blots hybridized for the *MEA-ISR* repeat using DNA digested with the methyl-sensitive restriction enzyme *Msp*I. (D) Graphical representation of bisulfite sequencing of *MEA-ISR* with % cytosine methylation shown for each genotype. Error bars represent 95% confidence limits (Wilson score interval). CG sequence contexts are shaded dark grey, CHG are shaded light grey and white represents CHH.

DRM2 and DRM3 show a similar domain organization of N-terminal UBA domains and a C-terminal cytosine methyltransferase domain (Figure 1A). To further understand DRM2 function we performed site-directed mutagenesis of these domains and tested the consequence on RNA–directed DNA methylation. Cytosine methyltransferases require a set of 10 conserved motifs, including motif IV which contains the catalytic cysteine involved in transfer of the methyl group to cytosine (Figure 1B and 1C) [13]. We substituted the DRM2 motif IV catalytic cysteine C587 for an alanine, within an otherwise complementing *DRM2* transgene containing a Myc-epitope translational fusion (hereafter termed *DRM2_cat_-Myc*) (Figure 4A) [58]. DRM proteins also possess N-terminal UBA domains, which consist of three helices connected by two conserved loop regions forming an ubiquitin interaction surface [63], [64]. The first loop contains a highly conserved MGF/MGY motif, which is required for correct folding and maintenance of UBA domain structure [64]. To test the functional importance of the DRM2 UBA domains we substituting conserved phenylalanine residues (F73, F123, F206) in each loop-I region to alanines (hereafter termed *DRM2_uba_-Myc*) (Figure 4A). The wild type *DRM2-Myc* and mutant *DRM2_cat_-Myc* and *DRM2_uba_-Myc* transgenes were transformed into *drm1 drm2* and tested for their ability to complement mutant RNA–directed DNA methylation phenotypes.

We first tested whether the catalytic and UBA mutations introduced into *DRM2* had any consequence on protein accumulation and localization. Intact *DRM2-Myc drm1 drm2* nuclei immunostained using α-Myc antibodies show signal throughout the nuclei with numerous bright foci and exclusion from the strongly DAPI-staining chromocentres (Figure 4B) [53]. The DRM2_cat_-Myc and DRM2_uba_-Myc mutant proteins showed comparable staining patterns to those observed for the unmutated DRM2-Myc protein (Figure 4B). This indicates that the UBA and methyltransferase mutations introduced do not cause a defect in DRM2 protein stability or localization to the nucleus.

We next tested the ability of the *DRM2* transgenes to complement *drm1 drm2* by assaying maintenance of non-CG methylation at *MEA-ISR* using methyl-sensitive *Msp*I digestion followed by Southern blotting and hybridization as described earlier. The wild type *DRM2-Myc* transgene complemented *drm1 drm2*, whereas the *DRM2_cat_-Myc* and *DRM2_uba_-Myc* lines showed a complete and partial failure to remethylate *MEA-ISR* respectively (Figure 4C). We confirmed these observations by sequencing *MEA-ISR* following sodium bisulfite conversion; non-CG methylation was restored by *DRM2-Myc* but not by *DRM2_cat_-Myc*, whereas the *DRM2_uba_-Myc* transformants showed a low level of non-CG methylation, comparable to the level observed in *drm3-1* (Figure 2B, Figure 4D, Table S1 and Figure S3). Therefore, intact UBA and methyltransferase domains are required for DRM2 function during maintenance of non-CG DNA methylation.

**Figure 5:**
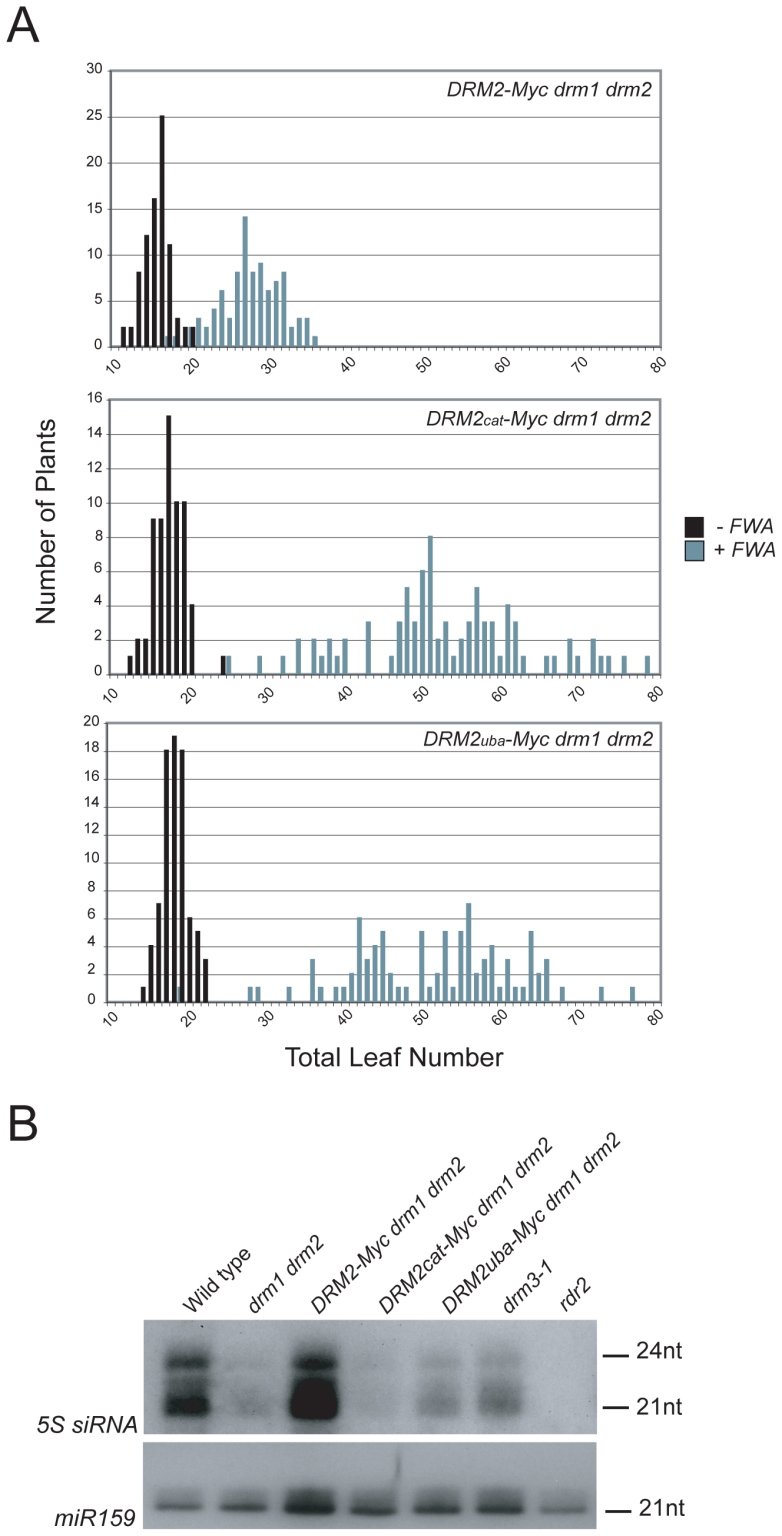
DRM2 catalytic and UBA domains are required for *de novo* DNA methylation and siRNA accumulation. (A) Histograms showing flowering-time distribution of untransformed plants (black bars) or T_1_ *FWA* transformant plants (grey bars). Flowering-time is measured as total leaf number and plotted along the *x*-axis, with number of plants on *y*-axis. (B) Northern blotting and hybridization of *5S rDNA* and *microRNA159* (*miR159*) small RNAs.

To test whether the *DRM2* mutations also blocked establishment of DNA methylation we transformed *DRM2-Myc drm1 drm2*, *DRM2_cat_-Myc drm1 drm2* and *DRM2_uba_-Myc drm1 drm2* with *FWA* and used the flowering-time distribution within T_1_ populations as a measure of silencing. The *FWA* T_1_ populations generated in *DRM2_cat_-Myc drm1 drm2*, *DRM2_uba_-Myc drm1 drm2* backgrounds flowered significantly later than the wild type *DRM2-Myc drm1 drm2* control population (Figure 5A and Table 1). We interpret this as meaning that intact UBA and methyltransferase domains are also required to establish RNA–directed DNA methylation and silencing at *FWA*.

Although *DRM2* is targeted by siRNAs, it is also required for accumulation of specific siRNA populations, for example siRNAs homologous to the *5S* ribosomal DNA genes [29], which may reflect a feedback loop between transcriptional silencing and siRNA biogenesis. *5S* siRNAs detected by northern blotting and hybridization are absent in *rdr2* and greatly reduced in *drm1 drm2*, relative to wild type (Figure 5B) [29]. The *DRM2-Myc* transgene is able to restore *5S* siRNA accumulation in the *drm1 drm2* mutant, but this complementation is not evident with either *DRM2_cat_-Myc* or *DRM2_uba_-Myc* (Figure 5B). Consistent with *DRM3* functioning to promote DRM2 activity, we also observed a strong reduction in the accumulation of *5S* siRNAs in *drm3-1* mutants (Figure 5B). Together these data demonstrate that both the DRM2 catalytic methyltransferase and UBA domains are required for establishment and maintenance of RNA–directed DNA methylation *in vivo*.

## Discussion

A complex pathway of genes has been defined that generates siRNAs homologous to repeated sequences, which can guide DRM2 cytosine methyltransferase activity to target DNA sequences [16]–[34]. Here we define a new member of this pathway, the catalytically mutated DNA cytosine methyltransferase paralog DRM3, which is required for normal maintenance of non-CG DNA methylation, establishment of RNA–directed DNA methylation triggered by tandem repeats and accumulation of repeat associated siRNAs. As *drm3* phenotypes are weaker than *drm2* we believe that DRM3 acts to promote activity of DRM2, but is not an absolute requirement. The strength of *drm3* phenotypes resembles those observed for *dcl3*, which is defective in accumulation of 24 nt siRNA known to promote RNA–directed DNA methylation. As the *drm3-1 dcl3-1* double mutant shows an enhanced phenotype to the level of *drm2*, this suggests that the combined loss of 24 nt siRNA and DRM3 severely hinders DRM2 activity. The *drm3* mutation does not influence *DRM2* expression or protein accumulation, leading us to believe that DRM3 acts together with DRM2 at the step of catalysis. Indeed, several examples exist of catalytically inactive paralogs acting to stimulate the activity of catalytic partners [65], [66]. In this respect the stimulatory interaction between Dnmt3a and Dnmt3L is particularly notable [42]–[46]. Despite extensive testing we have been unable to find *in vitro* conditions where *A.thaliana* DRM2 is active as a cytosine methyltransferase, which has precluded us testing a direct stimulatory effect for DRM3 on catalysis. Development of these assays will be an important next step to dissect the function of DRM2 and DRM3 during RNA–directed DNA methylation.

Although an intriguing parallel exists between the functional relationships of DRM2/DRM3 and Dnmt3a/Dnmt3L, phylogenetic analysis strongly suggests that these protein families diverged independently in plants and animals (Figure 1A). There are also important differences between DRM and Dnmt3 in the mechanism of recruitment to genomic target sites. The Dnmt3L protein plays a key role in targeting Dnmt3 via an N-terminal PHD domain, which specifically recognizes histone 3 unmethylated at lysine 4 [41]–[46]. By analogy the N-terminal UBA domains of DRM2 may be involved in targeting DNA methyltransferase activity. Supporting this idea we have shown that the DRM2 UBA domains are critical for RNA directed DNA methylation. We do not currently know whether DRM3 UBA domains are also functionally important. Sequence analysis shows that all DRM3 proteins possess two UBA domains, the second of which contains conserved residues known to be important for proper domain folding, including the MGF/MGY motif in loop-I (Figure S2) [64]. Interestingly, the first UBA domain of DRM3 proteins show substitution of the MGF/MGY glycine for either lysine or asparagine, which would be predicted to abolish proper folding [64]. However, as UBA domains are able to bind ubiquitin independently, DRM3 UBA domains may still play a functional role [63], [64]. The precise functions of the DRM UBA domains are currently unknown, though it is interesting to speculate that they may recognize specific chromatin modifications during RdDM.

In mammals PIWI proteins together with interacting piRNA act upstream of Dnmt3a/Dnmt3L to DNA methylate homologous repeats during germ line development [40], [42]–[48]. Plants lack orthologs of the PIWI proteins and have not so far been observed to produce piRNA [67]. Thus, small RNA–directed DNA methylation pathways appear to have evolved independently in plants and animals. Equally, the specific rearrangement of methyltransferase catalytic motifs shared between all DRM proteins indicates that DRM and Dnmt3 families have diverged independently in plants and animals (Figure 1). Here we demonstrate a further example of convergence in the parallel evolution of catalytically mutated cytosine methyltransferase paralogs (DRM3 and Dnmt3L) in both lineages. It is also important to note that information other than small RNAs must be involved in targeting DNA methylation, as this mark can be established and maintained in *Neurospora crassa* RNAi mutants [53], and no evidence exists of small RNAs acting upstream of Dnmt3a in the mammalian female germ line [48]. In conclusion, comparison of DRM3 and Dnmt3L function provides an example of mechanistic convergence between plant and mammalian silencing systems.

## Materials and Methods

### Plant materials

The *drm3-1* allele is T-DNA insertion Salk_136439 and *drm3-2* allele is T-DNA insertion Sail_1215_B08. The presence of the *drm3-1* insertion can be genotyped by PCR amplification using JP3192 together with a primer to the T-DNA left border JP2410 (all oligonucleotide sequences are listed in Table S4). The presence of the *drm3-2* insertion can be genotyped using primers JP3510 and JP2027. The *drm1 drm2* and *dcl3-1* T-DNA insertions have been described previously [7], [16]. *DRM3* complementation was performed by PCR amplification of an 8kb *DRM3* genomic region from Columbia DNA using oligonucleotides JP3477 and JP3479 and cloned into pCR2.1 (Invitrogen). The *DRM3* genomic region then was cloned into the pCAMBIA1300 binary vector as a *Sal*I restriction fragment and used to transform *drm3-1* plants by floral dipping using *Agrobacterium* strain ASE. The *DRM2-Myc* transgene has been described previously [58]. This construct was mutagenized using Quikchange (Stratagene) using oligonucleotides JP603/JP604 (catalytic mutation C587A), JP2631/JP2632 (UBA domain 1 F73A), JP2633/JP2634 (UBA domain 2 F123A) and JP2635/JP2636 (UBA domain 3 F206A).

### Phylogenetic tree construction

Multiple sequence alignment was performed using the European Bioinformatics Institute CLUSTALW server (http://www.ebi.ac.uk/clustalw/). NCBI accession numbers or gene numbers for the proteins analyzed are (*Arabidopsis thaliana*) AtDRM1 At5g15380, AtDRM2 At5g14620, AtDRM3 At3g17310, (*Populus trichocarpa*) PtDRM2 DMT905, PtDRM3 DMT907, (*Oryzae sativa*) OsDRM2 Os3g02010, OsDRM3 Os5g04330, (*Homo sapiens*) HsDnmt3a NP_783328, HsDnmt3b CAB53071, HsDnm3L BAA95556, (*Mus musculus*) MmDnmt3a O88508, MmDnmt3b CAM27225, MmDnmt3L AAH83147 and (*Haemophilus parahaemolyticus*) HhaI P05102. The phylogenetic tree was constructed using the MEGA2 program using the neighbor-joining method. Bootstrap values were calculated with 1,000 replicates.

### Analysis of DNA methylation

Southern blotting and hybridization were performed using 1µg of genomic DNA digested overnight using *Msp*I and separated by gel electrophoresis using 0.8% agarose and blotted onto N+Hybond (Amersham) nitrocellulose. The *MEA-ISR* probe was amplified using primers JP980 and JP981. 1µg of genomic DNA was converted using a Methyleasy kit (Human Genetic Signatures) and *MEA-ISR* amplified using primers JP1026 and JP1027, corresponding to positions 53456–53602 of BAC clone T14P4 [15], [36]. PCR products were cloned using the TOPO TA cloning kit (Invitrogen) and sequenced using the T7 primer. The *FWA* promoter region bisulfite sequenced corresponds to co-ordinates 46062 to 46551 of BAC clone M7J2. This region was amplified using primers JP2004 and JP2005 followed by nested amplification with primers JP2004 and JP4423. During analysis of sodium bisulfite sequencing data the percentage of methylated sites across all clones were calculated for each sequence context. The Wilson score interval was used to find the 95% confidence intervals for these percentages. Percentages were compared with the appropriate wild type percentage using the Pearson chi square test (Table S1 and Table S2). To compensate for multiple testing using the Bonferroni adjustment, a *p*-value of 0.002 was used as the threshold for statistical significance. To analyze cytosine methylation at *AtSN1* genomic DNA was digested with *Hae*III in 40 µl alongside controls without restriction endonuclease. Samples were then analyzed by quantitative real-time PCR amplifying with primers JP6699 and JP6700 combined with Biorad SYBR Green Supermix using a MX3000 Stratagene cycler. A ratio was then calculated between the amount of DNA amplified for each sample with and without *Hae*III digestion.

### *FWA* transformation and flowering-time analysis

Plants were transformed via floral dipping using *Agrobacterium tumefaciens* strain ASE carrying a genomic *FWA* clone in the binary vector pCAMBIA1300. T_1_ transformant seed was selected by germination and growth on MS plates containing 50µg/ml hygromycin followed by transfer to soil under continuous light. Vegetative and cauline leaves were counted until production of the first flowers as a measure of flowering-time. Untransformed plants were grown alongside as controls.

### RNA analysis

1 gram of floral buds were used to extract and purify small RNAs as described previously [60]. Small RNAs were separated by gel electrophoresis, blotted onto nitrocellulose membrane and hybridized with end-labeled oligonucleotide probes miR159 (5′-TAGAGCTCCCTTCAATCCAAA-3′) and *5S* siRNA (5′-ATGCCAAGTTTGGCCTCACGGTCT-3′) [61]. End-labeling was performed using T4 polynucleotide kinase (New England Biolabs) and γ-32P-ATP (Amersham). To analyze mRNA accumulation total RNA was extracted using Trizol (Invitrogen) and converted to cDNA as described previously [53]. *DRM2* expression was analyzed using qRT-PCR with primers JP6069 and JP6070 and *DRM3* analyzed by RT-PCR using primers JP3192 and JP3193.

### Immunohistochemistry

Immunostaining of fixed nuclei was performed as previously reported [68].

## Supporting Information

Figure S1 DRM2 and DRM3 are expressed independently of one another. (A) Schematic diagram of At3g17310 (DRM3) with exons indicated by open boxes. The position of T-DNA insertions in the *drm3-1* and *drm3-2* alleles is indicated by open triangles. RNA was extracted from wild type (Columbia), *drm1 drm2* and *drm3-1* and used to generate cDNA. The expression of DRM3 was tested by amplifying from this cDNA using primers JP3192 and JP3193 that span the insertion site in *drm3-1*. UBIQUITIN expression was analyzed as a control using amplification with primers JP3483 and JP3484. (B) Quantitative RT-PCR was used to measure mRNA expression levels of DRM2 (dark grey) and ACTIN (light grey) in wild type (Col) and *drm3-1*. (C) Western blotting detection of DRM2-Myc accumulation probing using α-Myc antibodies. Loading was analyzed by re-probing using α-CRY1 antibodies.
(0.65 MB PDF)

Figure S2 Sequence analysis of DRM UBA domains. UBA domains are typically ∼40 amino acids in length and form triple helical bundles [T. D. Mueller, J. Feigon, J Mol Biol 319, 1243 (Jun 21, 2002)]. Hydrophobic patches on the UBA surface are believed to stabilize interaction with hydrophobic surface of five-stranded ubiquitin β-sheet [T. D. Mueller, J. Feigon, J Mol Biol 319, 1243 (Jun 21, 2002)]. The most conserved residues are the MGF/MGY loop which connects helix 1 and 2 and is required for ubiquitin interaction [T. D. Mueller, J. Feigon, J Mol Biol 319, 1243 (Jun 21, 2002)]. The figure shows sequence alignments of DRM protein UBA domains performed using clustalW. Sequences are annotated with reference to solution structures of UBA domains and the presence of conserved residues [T. D. Mueller, J. Feigon, J Mol Biol 319, 1243 (Jun 21, 2002)]. The approximate helix regions are indicated above the sequences by black arrows and joining loop regions indicated by blue arrows. Conserved amino acids are highlighted in colour according to their side-chain; green indicates small (G, A), purple indicates hydrophobic (M, I, L, V), red indicates aromatic (F, Y) and blue indicates amide or acidic (K, N). AtDRM2 is exceptional in possessing three N-terminal UBA domains, whereas all other DRM proteins analyzed possess two recognizable UBA domains. In all DRM proteins the second UBA domain shows the presence of a conserved MGF motif within loop-I and the presence of other conserved hydrophobic residues. However, the first UBA domains in DRM3 proteins differ in that the conserved glycine in loop-I is replaced by either a lysine or asparagine. Mutation of this glycine in other UBA domains is sufficient to abolish interaction with ubiquitin [T. D. Mueller, J. Feigon, J Mol Biol 319, 1243 (Jun 21, 2002)]. Hence, it is possible that the first UBA domain in DRM3 proteins is inactive. However, as UBA domains have been shown to bind ubiquitin independently within a protein [T. D. Mueller, J. Feigon, J Mol Biol 319, 1243 (Jun 21, 2002)], it is possible that the second DRM3 UBA domain is functional.
(0.64 MB PDF)

Figure S3 Sodium bisulfite sequencing at *MEA-ISR* and *FWAM*. Graphical representation of sodium bisulfite analysis of the *MEA-ISR* and *FWA* tandem repeats. Sequences are shown with cytosines colored according to their sequence context; CG is blue, CHG is red and CHH is orange. Above the sequence are stacked blocks, each row of which represents an independent sequencing read. Methylation detected in these reads is represented by shading, again colored according to sequence context with CG blue, CHG red and CHH orange. The genotype in each case is stated at the top of each diagram.
(0.58 MB PDF)

Table S1 Sodium bisulfite sequencing analysis of *MEA-ISR*. The region analyzed corresponds to positions 53456 to 53602 of BAC clone T14P4. The *MEA-ISR* repeat was amplified from sodium bisulfite converted DNA and the frequency of cytosine versus thymine scored. The number of independent clones analyzed is indicated, together with the number of cytosine sites scored, the number observed to be methylated and the methylation frequency expressed as a %. There are 9 CG sites, 2 CHG sites and 24 CHH sites in the amplified region. The 95% confidence limits are given by the Wilson score interval. The p-values are from Pearson chisquare tests comparing each sample with wild type (Col). In addition, *drm3-1* was compared with *drm1 drm2* for CG (*p*-value = 0.28), CHG (*p*-value = 0.31) and CHH (*p*-value = 2.9×10^−5^) sites. Although the percentages for Col, *drm3-1* and *drm1 drm2* are similar between CHG and CHH methylation, the statistical power of comparisons between the three genotypes is much lower for CHG methylation because of the smaller number of sites analyzed in the sequenced region. Hence, the confidence intervals overlap and the statistical tests fail to reach significance for the CHG context. However, the same pattern is observed for the FWA locus (Table S2), where the difference between Col and *drm3-1* is statistically significant. In addition, Southern blotting and hybridization for *MEA-ISR* following *MspI* digestion (Figure 2A) measures methylation at the second CHG site and yields supporting observations of CHG methylation being intermediate in *drm3-1*, between wild type and *drm1 drm2*.
(0.05 MB DOC)

Table S2 Sodium bisulfite sequencing analysis of *FWA*. The region analyzed corresponds to co-ordinates 46062 to 46551 of BAC clone M7J2. The *FWA* repeats were amplified from sodium bisulfite converted DNA and the frequency of cytosine versus thymine scored. The number of independent clones analyzed is indicated, together with the number of cytosine sites scored, the number observed to be methylated and the methylation frequency expressed as a %. There are 12 CG sites, 10 CHG sites and 41 CHH sites in the amplified region. The 95% confidence limits are given by the Wilson score interval. The *p*-values are from Pearson chisquare tests comparing each sample with wild type (Col). In addition, *drm3-1* was compared with *drm1 drm2* for CG (*p*-value<0.007), CHG (*p*-value = <0.40) and CHH (*p*-value<0.002) sites.
(0.04 MB DOC)

Table S3 Average flowering-time of T_2_ *FWA* transformant lines.
(0.04 MB DOC)

Table S4 Oligonucleotide sequences.
(0.04 MB DOC)
