# Supplementary figures and images for: The De Novo Cytosine Methyltransferase DRM2 Requires Intact UBA Domains and a Catalytically Mutated Paralog DRM3 during RNA–Directed DNA Methylation in Arabidopsis thaliana
Source: PLoS Genet. 2010 Oct 28;6(10):e1001182. doi: 10.1371/journal.pgen.1001182 (PMC2965745; doi:10.1371/journal.pgen.1001182)

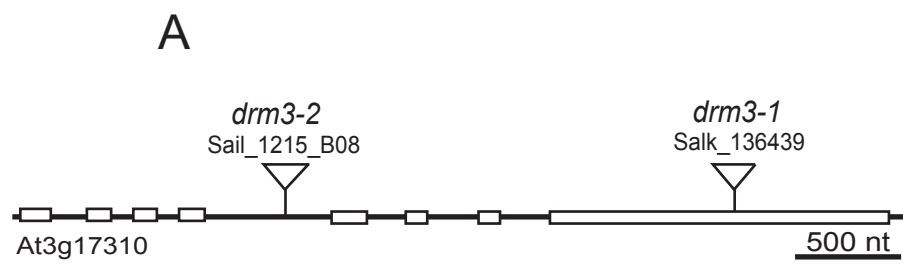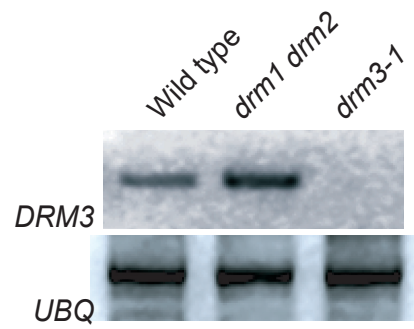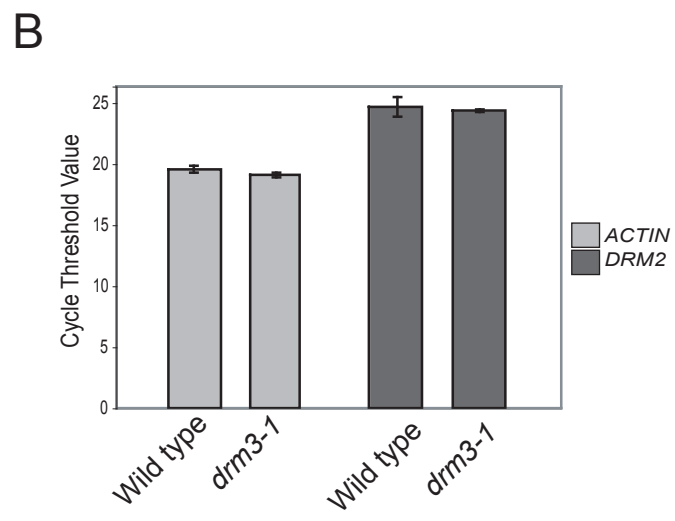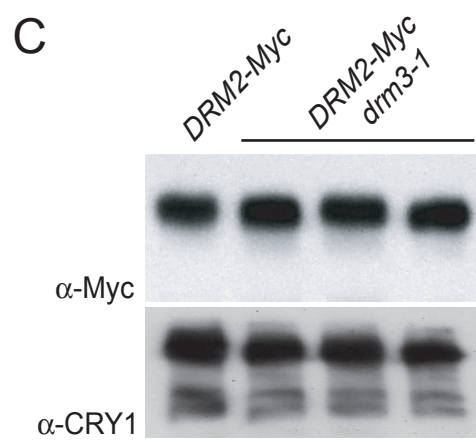

Supplement: Figure S1 — DRM2 and DRM3 are expressed independently of one another. (A) Schematic diagram of At3g17310 (DRM3) with exons indicated by open boxes. The position of T-DNA insertions in the drm3-1 and drm3-2 alleles is indicated by open triangles. RNA was extracted from wild type (Columbia), drm1 drm2 and drm3-1 and used to generate cDNA. The expression of DRM3 was tested by amplifying from this cDNA using primers JP3192 and JP3193 that span the insertion site in drm3-1. UBIQUITIN expression was analyzed as a control using amplification with primers JP3483 and JP3484. (B) Quantitative RT-PCR was used to measure mRNA expression levels of DRM2 (dark grey) and ACTIN (light grey) in wild type (Col) and drm3-1. (C) Western blotting detection of DRM2-Myc accumulation probing using α-Myc antibodies. Loading was analyzed by re-probing using α-CRY1 antibodies. (0.65 MB PDF) [file pgen.1001182.s001.pdf]
