# Supplementary material for: The De Novo Cytosine Methyltransferase DRM2 Requires Intact UBA Domains and a Catalytically Mutated Paralog DRM3 during RNA–Directed DNA Methylation in Arabidopsis thaliana
Source: PLoS Genet. 2010 Oct 28;6(10):e1001182. doi: 10.1371/journal.pgen.1001182 (PMC2965745; doi:10.1371/journal.pgen.1001182)
Supplement: Figure S2 — Sequence analysis of DRM UBA domains. UBA domains are typically ∼40 amino acids in length and form triple helical bundles [T. D. Mueller, J. Feigon, J Mol Biol 319, 1243 (Jun 21, 2002)]. Hydrophobic patches on the UBA surface are believed to stabilize interaction with hydrophobic surface of five-stranded ubiquitin β-sheet [T. D. Mueller, J. Feigon, J Mol Biol 319, 1243 (Jun 21, 2002)]. The most conserved residues are the MGF/MGY loop which connects helix 1 and 2 and is required for ubiquitin interaction [T. D. Mueller, J. Feigon, J Mol Biol 319, 1243 (Jun 21, 2002)]. The figure shows sequence alignments of DRM protein UBA domains performed using clustalW. Sequences are annotated with reference to solution structures of UBA domains and the presence of conserved residues [T. D. Mueller, J. Feigon, J Mol Biol 319, 1243 (Jun 21, 2002)]. The approximate helix regions are indicated above the sequences by black arrows and joining loop regions indicated by blue arrows. Conserved amino acids are highlighted in colour according to their side-chain; green indicates small (G, A), purple indicates hydrophobic (M, I, L, V), red indicates aromatic (F, Y) and blue indicates amide or acidic (K, N). AtDRM2 is exceptional in possessing three N-terminal UBA domains, whereas all other DRM proteins analyzed possess two recognizable UBA domains. In all DRM proteins the second UBA domain shows the presence of a conserved MGF motif within loop-I and the presence of other conserved hydrophobic residues. However, the first UBA domains in DRM3 proteins differ in that the conserved glycine in loop-I is replaced by either a lysine or asparagine. Mutation of this glycine in other UBA domains is sufficient to abolish interaction with ubiquitin [T. D. Mueller, J. Feigon, J Mol Biol 319, 1243 (Jun 21, 2002)]. Hence, it is possible that the first UBA domain in DRM3 proteins is inactive. However, as UBA domains have been shown to bind ubiquitin independently within a protein [T. D. Mueller, J. Feigon, [file pgen.1001182.s002.pdf]

```
AtDRM1 UBA-1    MMILETLFNYSASTEASSSKSKVINHFIAMGFPEEH--VIKAMQEHGDEDVGEITNALLT 148
AtDRM2 UBA-2    SVIIDTISKYSSDCEAGSSKSKAIDHFLAMGFDEEK--VVKAIQEHGEDNMEAIANALLS 149
PtDRM2 UBA-1    -----SSSAEASSSVGSSSGSKMIDHFVKMGFPEKMKMVAKAIKENGEGDADSVLETLLT 97
OsDRM2 UBA-1    -------LPQDANGKANGSG-ALVAEFMGMGFPKEM--ILKAIKEIGDTDTEQLLELLLT 90
AtDRM3 UBA-1    SFHGLMEPKPEPDIEYET-DRIRIALLT-MKFPENL--VDFALDRLGKDTPIDEMVDFIV 156
PtDRM3 UBA-1    KYSTVTGVKEEPDVFDEVYDDKRVSLLK-MNFPAKE--VELAMDKLGENAPINEIIDFII 173
OsDRM3 UBA-1    HFAPRKEVIQDIKVEADSSSEKRSYLLSTMNFSQRE--VDLALNQLGEEASLEQLVDFIV 169
```

```
AtDRM1 UBA-2    ---LSSDDEEDELNNSSNEDRILQALIKMGYLREDAAIAIERCGEDASMEEVVDFICAAQ 232
AtDRM2 UBA-3    M--LNSDDEKD--PNSNENGSKIRSLVKMGFSELEASLAVERCGENVDIAELTDFLCAAQ 235
PtDRM2 UBA-2    FSDVDSADDEVITKTVSDEDNKLAFLRRMGYKEADASIAITRCGTEATISELADFICAAQ 187
OsDRM2 UBA-2    TPHSDGSGDEDFFQEMSEKDEKMKSLVNMGFPEDEAKMAIDRC-LDAPVAVLVDSIYASQ 194
AtDRM3 UBA-2    --------PEVPNEQLFETMDKTLRLLEMGFSNDEISMAIEKIGTKGQISVLAESIVTGE 243
PtDRM3 UBA-2    SWHYDDYYQDVNDETLYGTMDKTLCLLNMGFSENEVSLAIDKFGSEVPVTELANAICAHQ 280
OsDRM3 UBA-2    ---------EVKDESLFGVMDKTLHLLQMGFTEEEVSSVIDKAGPEATVLELADTIFARR 238
```
