# Supplementary material for: The De Novo Cytosine Methyltransferase DRM2 Requires Intact UBA Domains and a Catalytically Mutated Paralog DRM3 during RNA–Directed DNA Methylation in Arabidopsis thaliana
Source: PLoS Genet. 2010 Oct 28;6(10):e1001182. doi: 10.1371/journal.pgen.1001182 (PMC2965745; doi:10.1371/journal.pgen.1001182)

MEA-ISR

Col

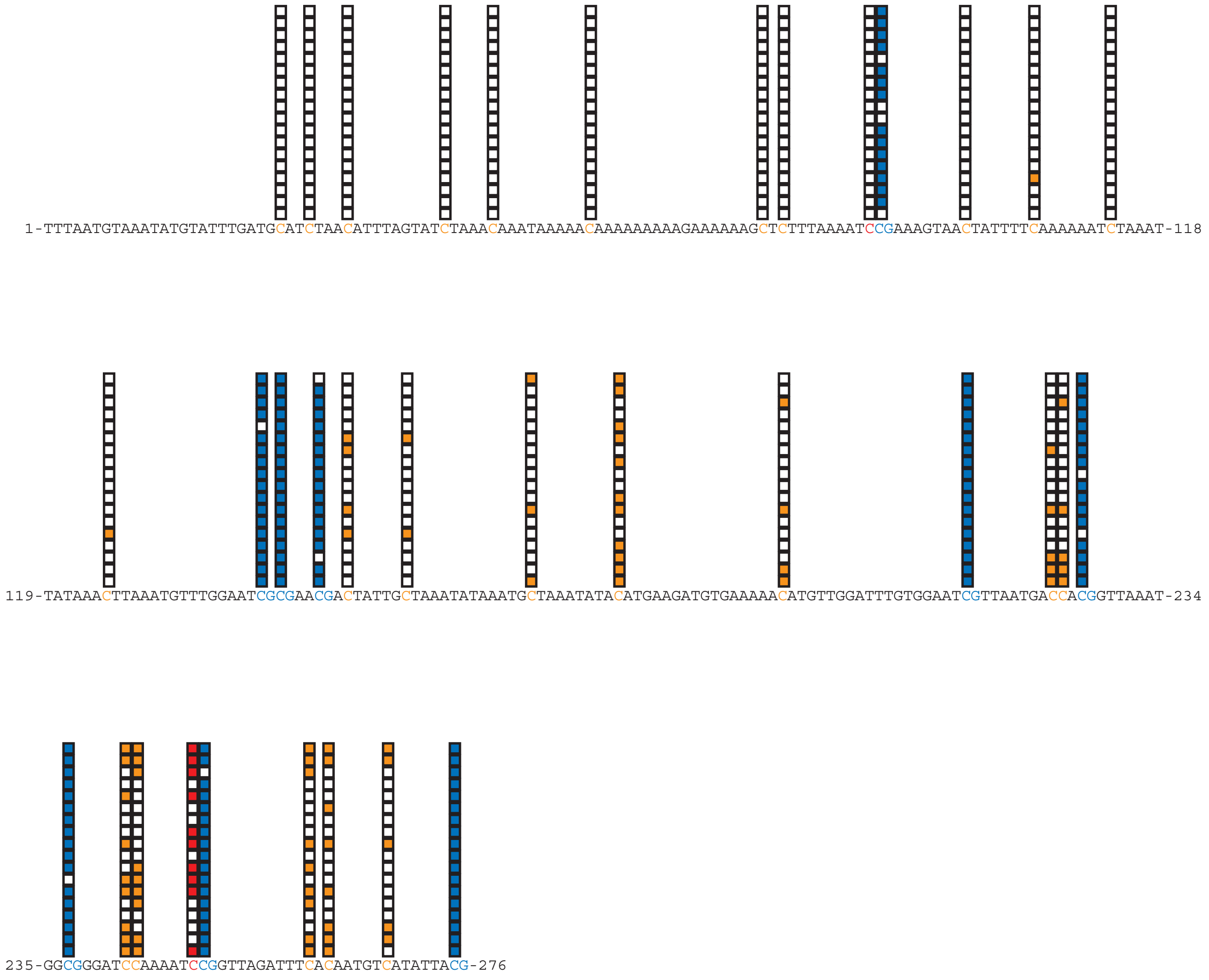
1-TTTAATGTAAATATGTATTTGATGCATCTAACATTTAGTATCTAAACAAATAAAAACAAAAAAAAAGAAAAAAGCTCTTTAAAATCCGAAAGTAACTATTTTCAAAAAATCTAAAT-118
119-TATAAACTTAAATGTTTGGAATCGCGAACGACTATTGCTAAATATAAATGCTAAATATACATGAAGATGTGAAAAACATGTTGGATTTGTGGAATCGTTAATGACCACGGTTAAAT-234
235-GGCGGGATCCAAAATCCGGTTAGATTTCACAATGTCATATTACG-276

MEA-ISR
DRM2-Myc drm1 drm2

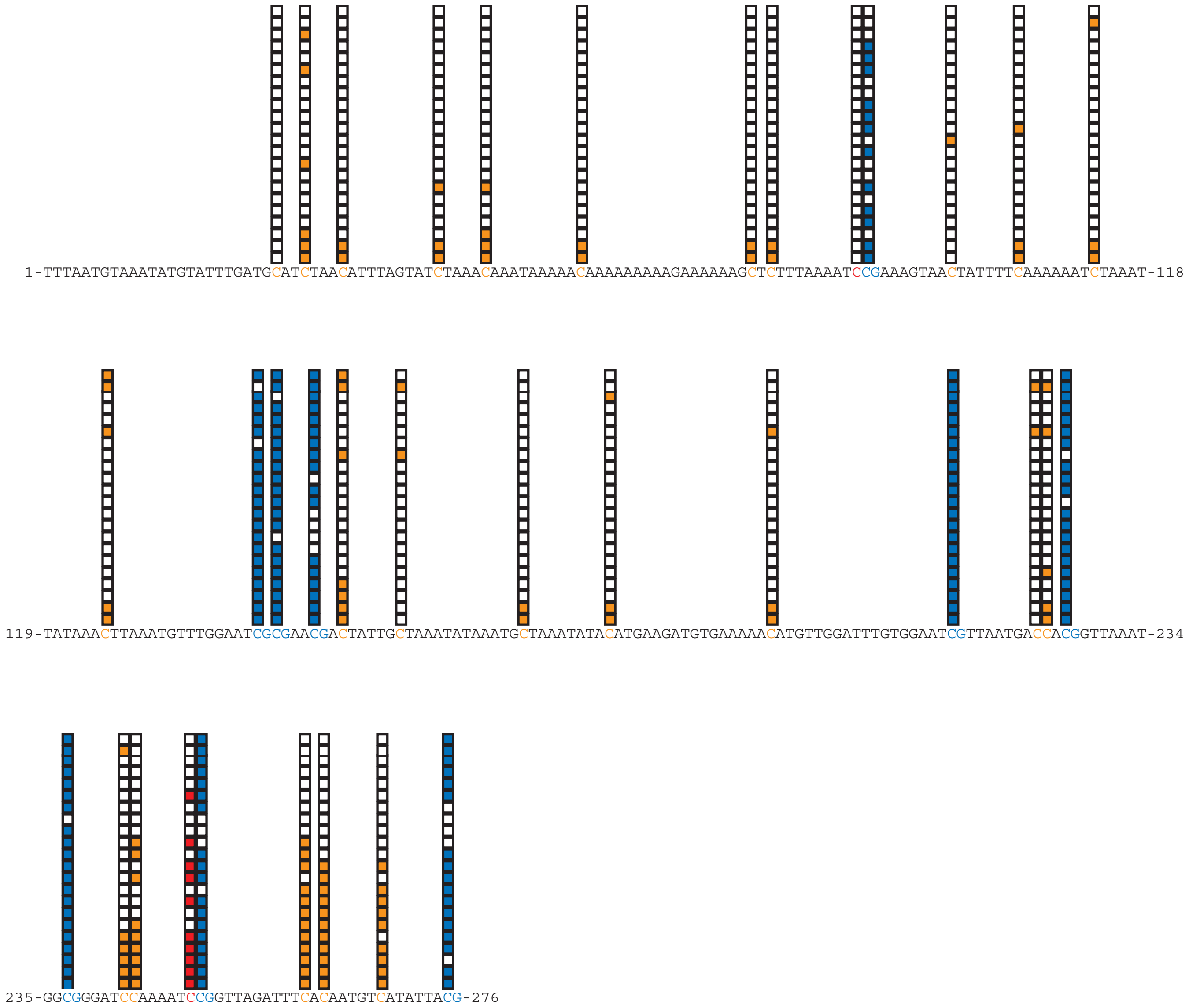

MEA-ISR

DRM2cat-Myc drm1 drm2

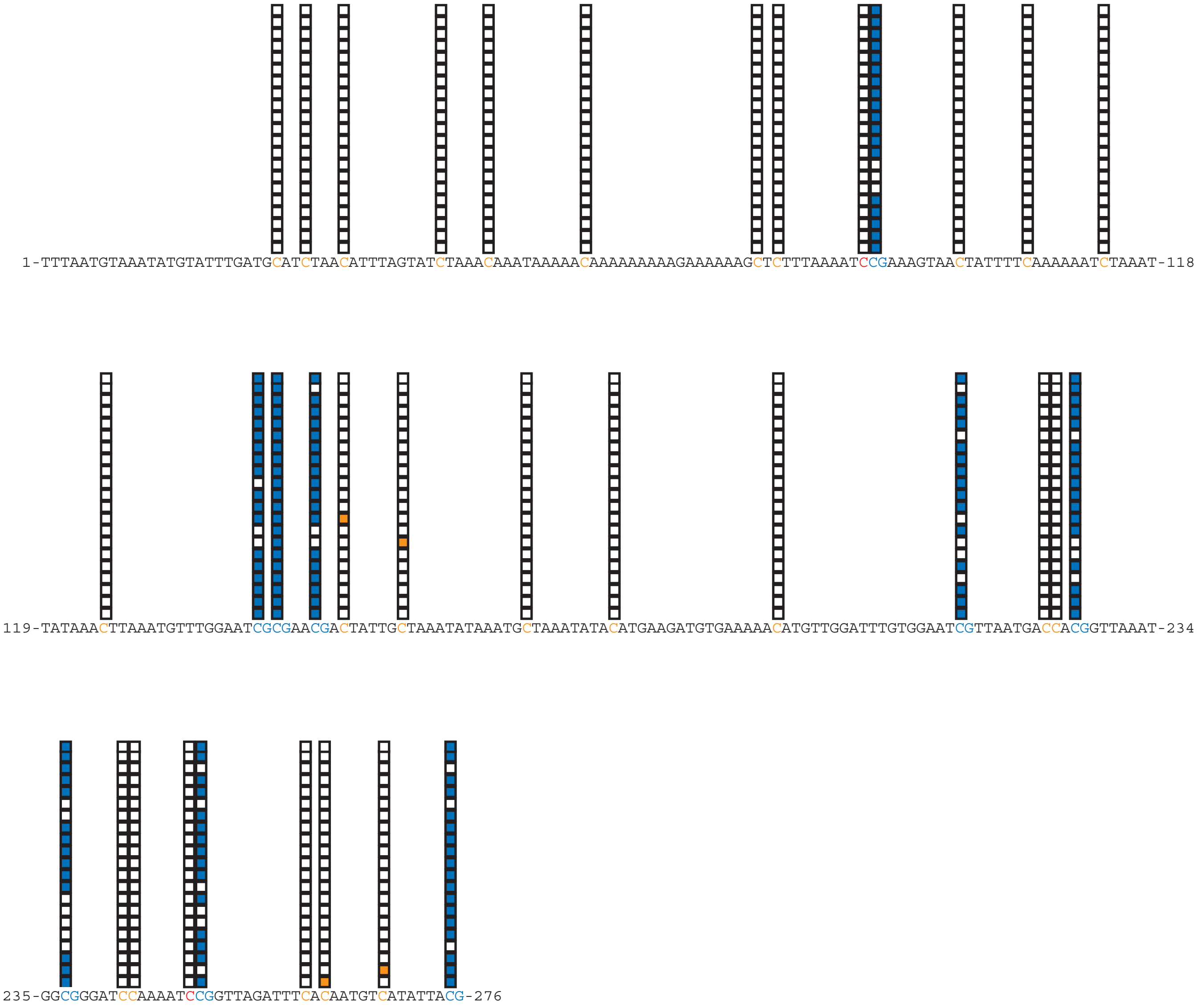

MEA-ISR
DRM2uba-Myc drm1 drm2

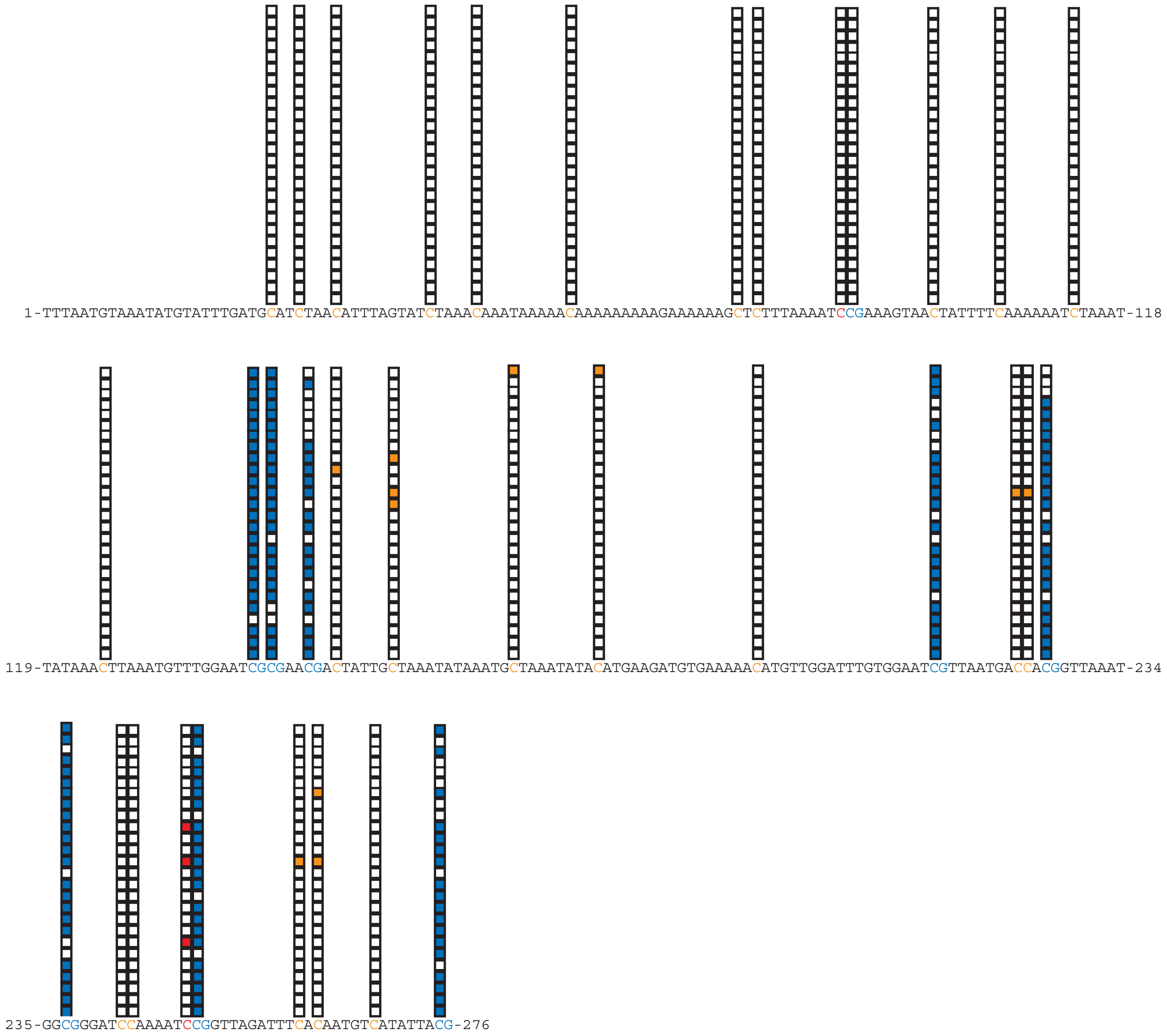

MEA-ISR
drm3-1

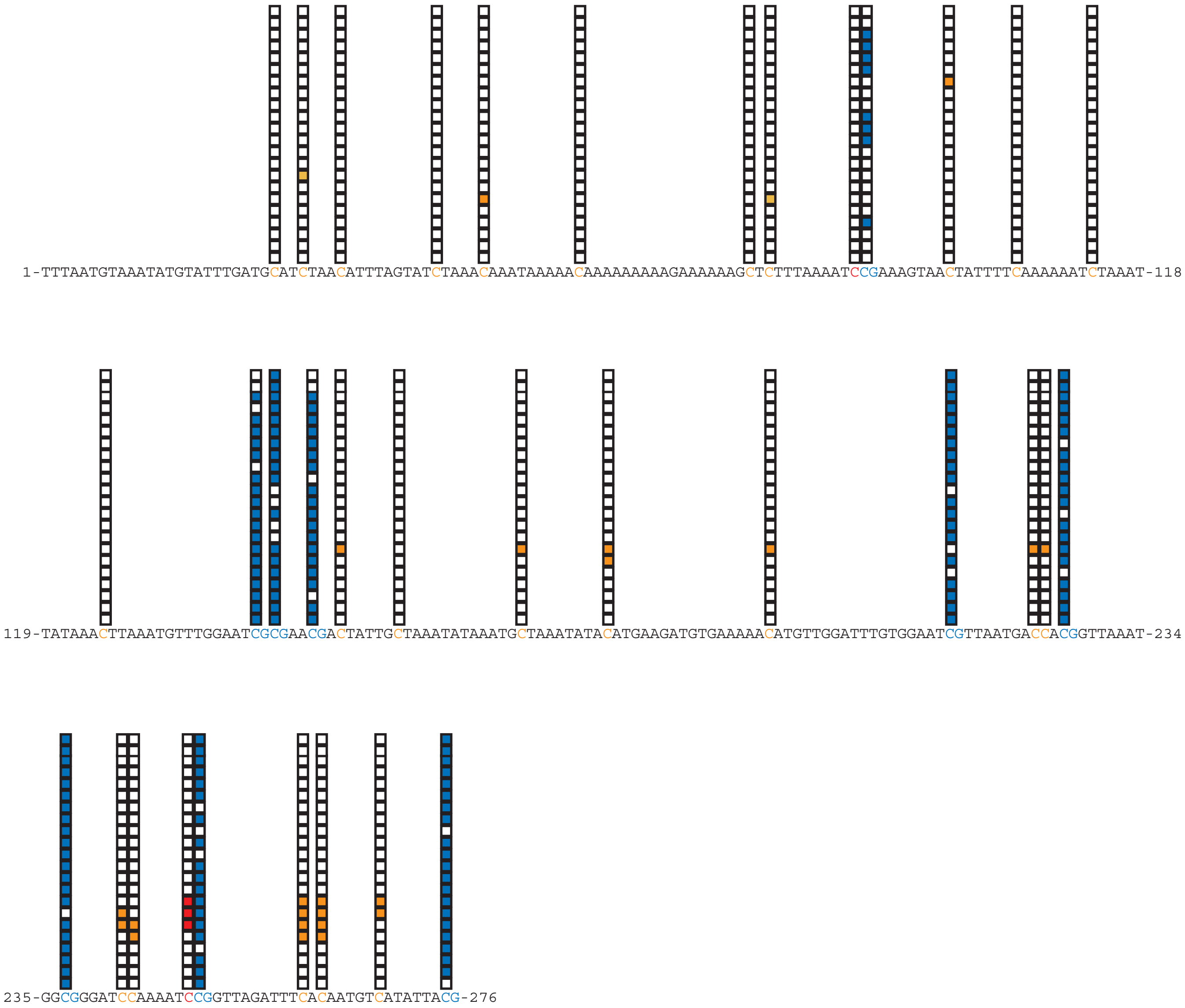

MEA-ISR
drm1 drm2

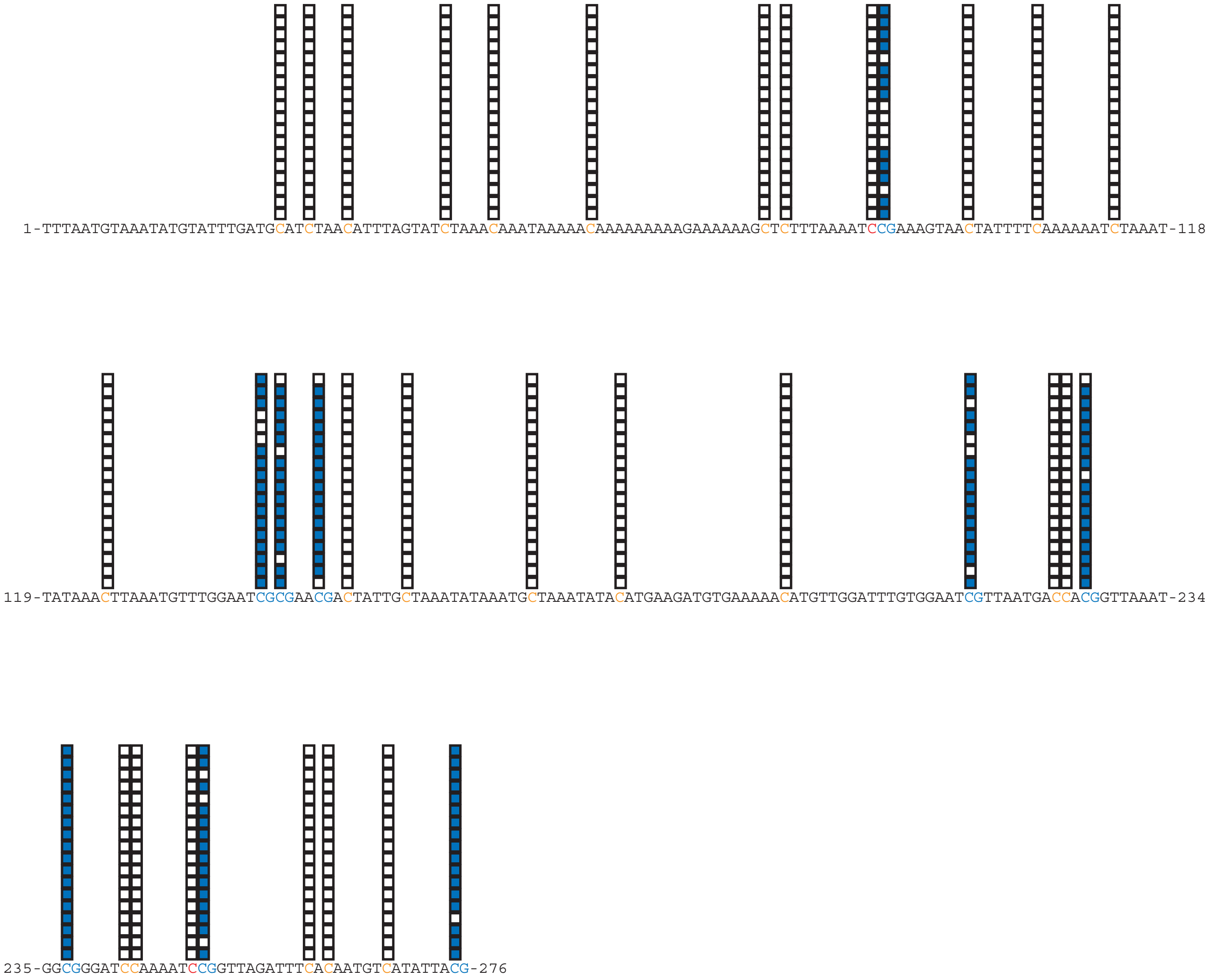

FWA

Col

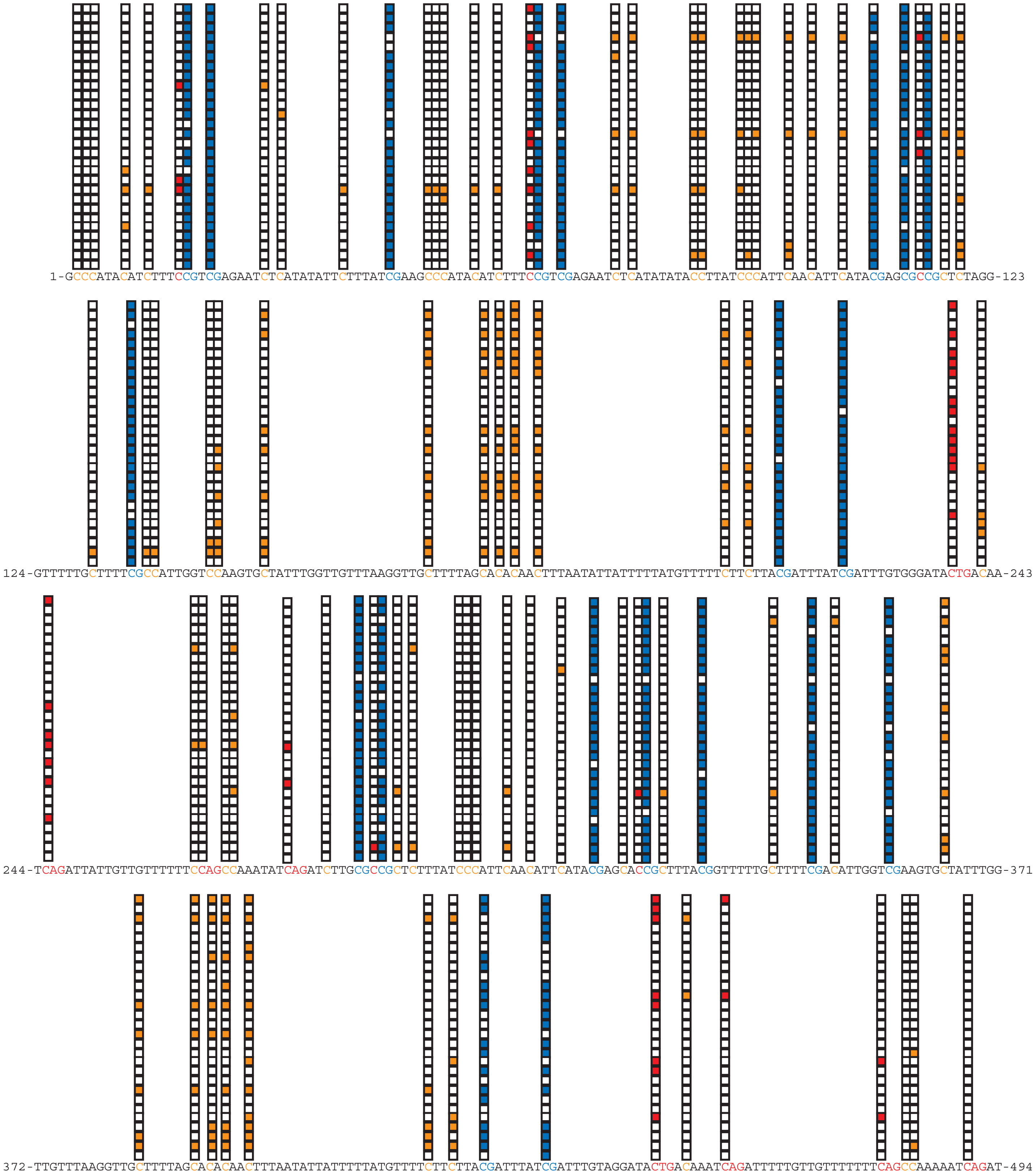
1-GCCCATACATCTTTCCGTCGAGAATCTCATATATTCTTTATCGAAGCCCATACATCTTTCCGTCGAGAATCTCATATATACCTTATCCCATTCAACATTCATACGAGCGCCGCTCTAGG-123
124-GTTTTTGCTTTTCGCCATTGGTCCAAGTGCTATTTGGTTGTTTAAGGTTGCTTTTAGCACACAACTTTAATATTATTTTTATGTTTTTCTTCTTACGATTTATCGATTTGTGGGATACTGACAA-243
244-TCAGATTATTGTTGTTTTTTCCAGCCAAATATCAGATCTTGCGCCGCTCTTTATCCCATTCAACATTCATACGAGCACCGCTTTACGGTTTTTGCTTTTCGACATTGGTCGAAGTGCTATTTGG-371
372-TTGTTTAAGGTTGCTTTTAGCACACAACTTTAATATTATTTTTATGTTTTCTTCTTACGATTTATCGATTTGTAGGATACTGACAAATCAGATTTTTGTTGTTTTTTTCAGCCAAAAATCAGAT-494

FWA

drm3-1

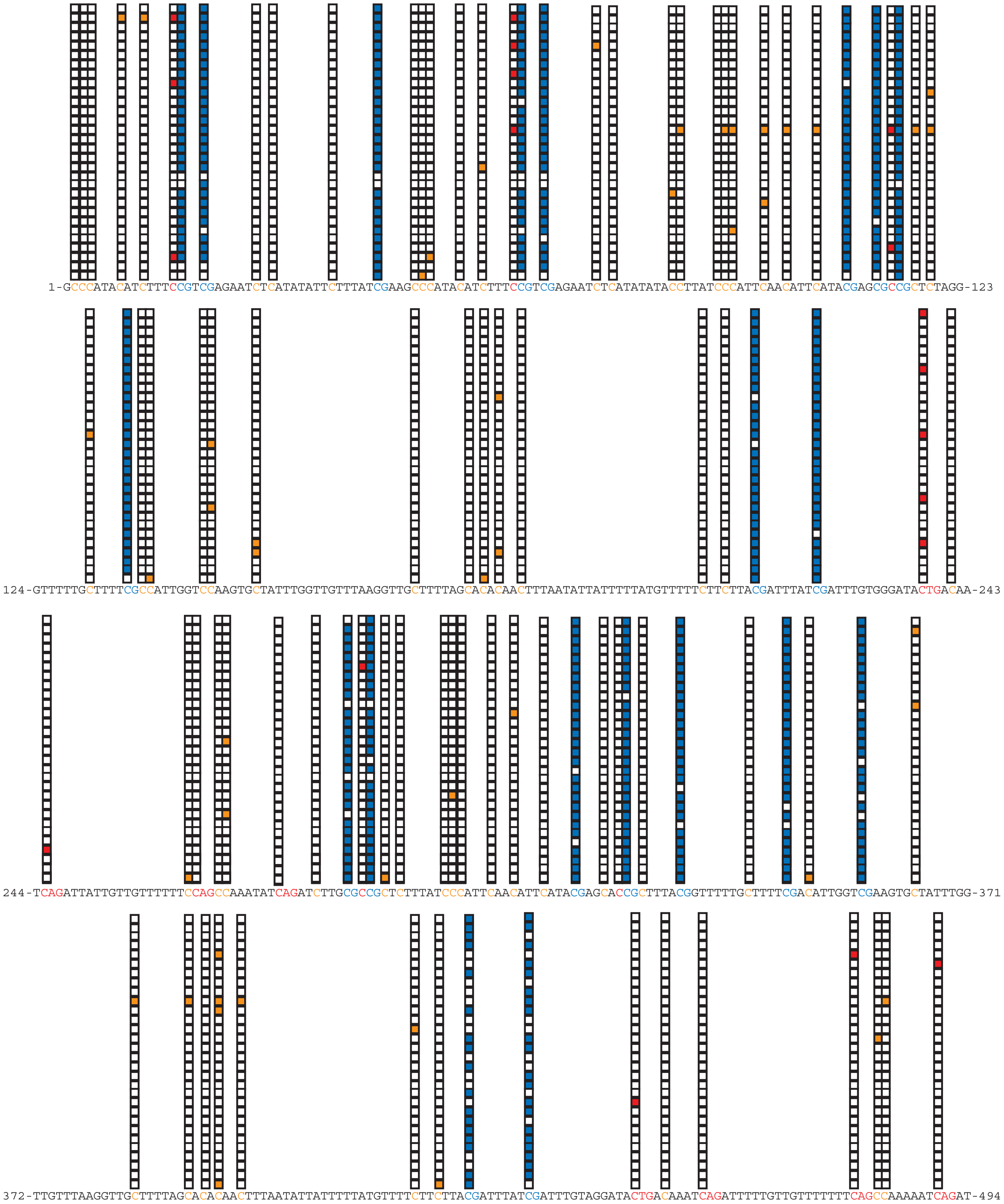
1-GCCCATACATCTTTCCGTCGAGAATCTCATATATTCTTTATCGAAGCCCATACATCTTTCCGTCGAGAATCTCATATATACCTTATCCCATTCAACATTCATACGAGCGCCGCTCTAGG-123
124-GTTTTTGCTTTTCGCCATTGGTCCAAGTGCTATTTGGTTGTTTAAGGTTGCTTTTAGCACACAACTTTAATATTATTTTTATGTTTTTCTTCTTACGATTTATCGATTTGTGGGATACTGACAA-243
244-TCAGATTATTGTTGTTTTTTCCAGCCAAATATCAGATCTTGCGCCGCTCTTTATCCCATTCAACATTCATACGAGCACCGCTTTACGGTTTTTGCTTTTCGACATTGGTCGAAGTGCTATTTGG-371
372-TTGTTTAAGGTTGCTTTTAGCACACAACTTTAATATTATTTTTATGTTTTCTTCTTACGATTTATCGATTTGTAGGATACTGACAAATCAGATTTTTGTTGTTTTTTTCAGCCAAAAATCAGAT-494

FWA

drm1 drm2

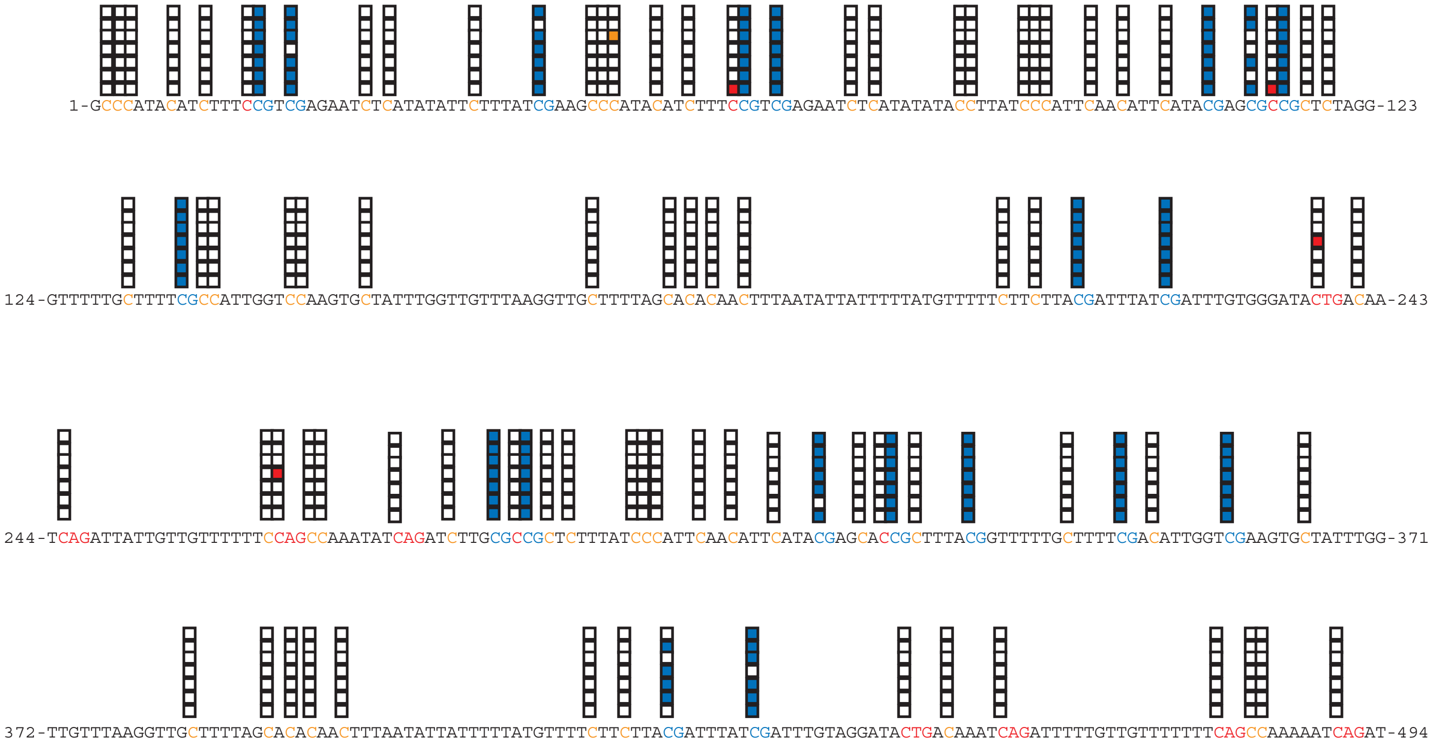

Supplement: Figure S3 — Sodium bisulfite sequencing at MEA-ISR and FWAM. Graphical representation of sodium bisulfite analysis of the MEA-ISR and FWA tandem repeats. Sequences are shown with cytosines colored according to their sequence context; CG is blue, CHG is red and CHH is orange. Above the sequence are stacked blocks, each row of which represents an independent sequencing read. Methylation detected in these reads is represented by shading, again colored according to sequence context with CG blue, CHG red and CHH orange. The genotype in each case is stated at the top of each diagram. (0.58 MB PDF) [file pgen.1001182.s003.pdf]
