# Supplementary material for: The De Novo Cytosine Methyltransferase DRM2 Requires Intact UBA Domains and a Catalytically Mutated Paralog DRM3 during RNA–Directed DNA Methylation in Arabidopsis thaliana
Source: PLoS Genet. 2010 Oct 28;6(10):e1001182. doi: 10.1371/journal.pgen.1001182 (PMC2965745; doi:10.1371/journal.pgen.1001182)
Supplement: Table S1 — Sodium bisulfite sequencing analysis of MEA-ISR. The region analyzed corresponds to positions 53456 to 53602 of BAC clone T14P4. The MEA-ISR repeat was amplified from sodium bisulfite converted DNA and the frequency of cytosine versus thymine scored. The number of independent clones analyzed is indicated, together with the number of cytosine sites scored, the number observed to be methylated and the methylation frequency expressed as a %. There are 9 CG sites, 2 CHG sites and 24 CHH sites in the amplified region. The 95% confidence limits are given by the Wilson score interval. The p-values are from Pearson chisquare tests comparing each sample with wild type (Col). In addition, drm3-1 was compared with drm1 drm2 for CG (p-value = 0.28), CHG (p-value = 0.31) and CHH (p-value = 2.9×10^−5^) sites. Although the percentages for Col, drm3-1 and drm1 drm2 are similar between CHG and CHH methylation, the statistical power of comparisons between the three genotypes is much lower for CHG methylation because of the smaller number of sites analyzed in the sequenced region. Hence, the confidence intervals overlap and the statistical tests fail to reach significance for the CHG context. However, the same pattern is observed for the FWA locus (Table S2), where the difference between Col and drm3-1 is statistically significant. In addition, Southern blotting and hybridization for MEA-ISR following MspI digestion (Figure 2A) measures methylation at the second CHG site and yields supporting observations of CHG methylation being intermediate in drm3-1, between wild type and drm1 drm2. (0.05 MB DOC) [file pgen.1001182.s004.doc]

# **Table S1.** Sodium bisulfite sequencing analysis of *MEA-ISR*.

| Genotype | Number of clones | Total CG sites | Methylated CG sites | % CG  methylation | 95% confidence interval | *P*-value |
| --- | --- | --- | --- | --- | --- | --- |
| Col  *DRM2-Myc drm1 drm2*  *DRM2cat-Myc drm1 drm2*  *DRM2uba-Myc drm1 drm2*  *drm3-1*  *drm1 drm2* | 18  22  21  26  22  18 | 162  198  189  234  198  162 | 155  167  154  164  159  138 | 95.7  84.3  81.5  70.1  80.3  85.2 | 91.4-97.9  78.6-88.8  75.3-86.4  63.9-75.6  74.2-85.2  78.9-89.8 | -  9.3x10^-4^  8.8x10^-5^  5.7x10^-10^  2.8x10^-5^  2.5x10^-3^ |
| Genotype | Number of clones | Total CHG sites | Methylated CHG sites | % CHG methylation |  |  |
| Col  *DRM2-Myc drm1 drm2*  *DRM2cat-Myc drm1 drm2*  *DRM2uba-Myc drm1 drm2*  *drm3-1*  *drm1 drm2* | 18  22  21  26  22  18 | 36  44  42  52  44  36 | 10  11  0  3  3  0 | 27.8  25.0  0.0  5.8  6.8  0.0 | 15.9-44.0  14.6-39.4  0.0-8.4  2.0-15.6  2.4-18.2  0.0-9.6 | -  9.7x10^-1^  9.1x10^-4^  1.1x10^-2^  2.6x10^-2^  2.2x10^-3^ |
| Genotype | Number of clones | Total CHH sites | Methylated CHH sites | % CHH methylation |  |  |
| Col  *DRM2-Myc drm1 drm2*  *DRM2cat-Myc drm1 drm2*  *DRM2uba-Myc drm1 drm2*  *drm3-1*  *drm1 drm2* | 18  22  21  26  22  18 | 432  528  504  624  528  432 | 80  104  4  11  23  0 | 18.5  19.7  0.8  1.8  4.4  0.0 | 15.1-22.5  16.5-23.3  0.3-2.0  1.0-3.1  2.9-6.5  0.0-0.9 | -  7.0x10^-1^  9.3x10^-21^  4.2x10^-21^  3.7x10^-12^  1.8x10^-20^ |
