# Supplementary material for: The De Novo Cytosine Methyltransferase DRM2 Requires Intact UBA Domains and a Catalytically Mutated Paralog DRM3 during RNA–Directed DNA Methylation in Arabidopsis thaliana
Source: PLoS Genet. 2010 Oct 28;6(10):e1001182. doi: 10.1371/journal.pgen.1001182 (PMC2965745; doi:10.1371/journal.pgen.1001182)
Supplement: Table S2 — Sodium bisulfite sequencing analysis of FWA. The region analyzed corresponds to co-ordinates 46062 to 46551 of BAC clone M7J2. The FWA repeats were amplified from sodium bisulfite converted DNA and the frequency of cytosine versus thymine scored. The number of independent clones analyzed is indicated, together with the number of cytosine sites scored, the number observed to be methylated and the methylation frequency expressed as a %. There are 12 CG sites, 10 CHG sites and 41 CHH sites in the amplified region. The 95% confidence limits are given by the Wilson score interval. The p-values are from Pearson chisquare tests comparing each sample with wild type (Col). In addition, drm3-1 was compared with drm1 drm2 for CG (p-value<0.007), CHG (p-value = <0.40) and CHH (p-value<0.002) sites. (0.04 MB DOC) [file pgen.1001182.s005.doc]

# **Table S2.** Sodium bisulfite sequencing analysis of *FWA*.

| Genotype | Number of clones | Total CG sites | Methylated CG sites | % CG  methylation | 95% confidence interval | *P*-value |
| --- | --- | --- | --- | --- | --- | --- |
| Col  *drm3-1*  *drm1 drm2* | 28  30  7 | 336  360  84 | 262  292  79 | 78.0  81.1  94.0 | 73.2-82.1  76.8-84.8  86.8-97.4 | 3.5x10^-1^  1.3x10^-3^ |
| Genotype | Number of clones | Total CHG sites | Methylated CHG sites | % CHG methylation |  |  |
| Col  *drm3-1*  *drm1 drm2* | 28  30  7 | 280  300  70 | 51  19  2 | 18.2  6.3  2.9 | 14.1-23.2  4.1-9.7  0.8-9.8 | 2.0x10^-5^  2.5x10^-3^ |
| Genotype | Number of clones | Total CHH sites | Methylated CHH sites | % CHH methylation |  |  |
| Col  *drm3-1*  *drm1 drm2* | 28  30  7 | 1148  1230  287 | 223  46  0 | 19.4  3.7  0 | 17.2-21.8  2.8-5.0  0-1.3 | 3.5x10^-33^  9.5x10^-16^ |
