# Supplementary material for: The De Novo Cytosine Methyltransferase DRM2 Requires Intact UBA Domains and a Catalytically Mutated Paralog DRM3 during RNA–Directed DNA Methylation in Arabidopsis thaliana
Source: PLoS Genet. 2010 Oct 28;6(10):e1001182. doi: 10.1371/journal.pgen.1001182 (PMC2965745; doi:10.1371/journal.pgen.1001182)
Supplement: Table S3 — Average flowering-time of T2 FWA transformant lines. (0.04 MB DOC) [file pgen.1001182.s006.doc]

# **Table S3.** Average flowering-time of T_2_ *FWA* transformant lines.

|  | Total leaf number  +/- standard error | |
| --- | --- | --- |
| Genotype and line number | T_1_ leaf number | T_2_ progeny |
| Wild type Col4-3  Col5-1  Col1-3  Col3-1  Col2-5 Col1-1Col3-8Col2-6 Col4-8  Col4-4  *drm3* 7-9  *drm3* 12-19  *drm3* 5-1  *drm3* 11-1  *drm3* 9-6  *drm3* 1-5  *drm3* 2-1  *drm3* 5-1  *drm3* 1-4  *drm3* 2-4  *drm1 drm2* 2-9  *drm1 drm2* 2-1  *drm1 drm2* 3-8  *drm1 drm2* 5-5  *drm1 drm2* 2-9  *drm1 drm2* 2-9 | 32  27  22  27  26  21  19  20  26  24  35  39  32  39  31  32  36  38  36  30  42  47  42  42  42  49 | 13.8 +/- 0.65  11.7 +/- 0.62  13.7 +/- 0.34  12.4 +/- 0.64  11.0 +/- 0.21  12.4 +/- 0.68  22.4 +/- 0.55  17.9 +/- 0.61  17.6 +/- 0.71  16.5 +/- 0.65  11.1 +/- 0.34  32.4 +/- 1.64  11.2 +/- 0.27  12.9 +/- 0.81  23.7 +/- 1.84  10.4 +/- 0.15  22.9 +/- 1.16  17.0 +/- 1.01  14.0 +/- 0.69  16.8 +/- 0.7  41.3 +/- 1.45  46.8 +/- 1.63  48.4 +/- 1.08  44.5 +/- 0.81  51.0 +/- 1.24  45.8 +/- 0.85 |
