# Supplementary material for: The De Novo Cytosine Methyltransferase DRM2 Requires Intact UBA Domains and a Catalytically Mutated Paralog DRM3 during RNA–Directed DNA Methylation in Arabidopsis thaliana
Source: PLoS Genet. 2010 Oct 28;6(10):e1001182. doi: 10.1371/journal.pgen.1001182 (PMC2965745; doi:10.1371/journal.pgen.1001182)
Supplement: Table S4 — Oligonucleotide sequences. (0.04 MB DOC) [file pgen.1001182.s007.doc]

# **Table S4.** Oligonucleotide sequences

| Name | Sequence |
| --- | --- |
| JP980  JP981  JP1026  JP1027  JP2027  JP2603  JP2604  JP2631  JP2632  JP2633  JP2634  JP2635  JP2636  JP3192  JP3193  JP3477  JP3479  JP3483  JP3484  JP3510  JP4549  JP4550  JP4551  JP4552  JP6063  JP6065  JP6067  JP6068  JP6069  JP6070  JP6699  JP6700  JP2004  JP2005  JP4423 | 5’-AAACCTTTCGTAAGCTACAGCCACTTTGTT-3’  5’-TCGGATTGGTTCTTCCTACCTCTTTACCTT-3’  5’-AAAGTGGTTGTAGTTTATGAAAGGTTTTAT-3’  5’-CTTAAAAAATTTTCAACTCATTTTTTTTAAAAAA-3’  5’- TAG CATCTGAATTTCATAACCAATCTCGATACAC-3’  5’-CTTGTAATTGGAGGAAGTCCTGCTAACAATCTGGCAGGCGGTAATAGG-3’  5’-CCTATTACCGCCTGCCAGATTGTTAGCAGGACTTCCTCCAATTACAAG-3’  5’-GTTTTTGCTACATTGTTTGACATGGGAGCTCCTGTTGAGATGATTTCTAGAGCGATC-3’  5’-GATCGCTCTAGAAATCATCTCAACAGGAGCTCCCATGTCAAACAATGTAGCAAAAAC-3’  5’-GCTATTGATCATTTCCTTGCTATGGGAGCTGATGAAGAAAAAGTTGTCAAAGCCATTC-3’  5’-GAATGGCTTTGACAACTTTTTCTTCATCAGCTCCCATAGCAAGGAAATGATCAATAGC-3’  5’-GCAAAATACGGTCTTTGGTGAAGATGGGTGCTTCAGAGCTTGAAGCTTCTTTAGCTGTC-3’  5’-GACAGCTAAAGAAGCTTCAAGCTCTGAAGCACCCATCTTCACCAAAGACCGTATTTTGC-3’  5’-AGAGATCAAGTGGTGTTGATGGG-3’  5’-TCCAGAAGAATACACTGGCTACG-3’  5’-GTCGACATCCTGATAGAATAGTTATATCTCAAGTTTATCACTGC-3’  5’-GTCGACATAACTTAAAACCTTATAATTAGATCAGATGTAAAACTTGTTCG-3’  5’-GATCTTTGCCGGAAAACAATTGGAGG-3’  5’-CGACTTGTCATTAGAAAGAAAGAGAT-3’  5’- TTAACATTAATAAACAACAGC-3’  5’-CGGGATCCATGGCTGATATGCGTAGACGAAATGG-3’  5’-CGGGATCCCTACGAACTTGAGTCACCCATATCTTCGG-3’  5’-CGGGATCCATGGAAACCCCATGGATGCAAGATGAG-3’  5’-CGGGATCCCTACATCATATCTCTGACACGTTTCG-3’  5’-CGGGATCCATGTACCCATACGACGTACCAGACTACGCAGTGATTTGGAATAACGATGATGATG  5’-CATGCCATGGTCAAGATCCTCTCATCCTCGCACG-3’  5’-AGGCGCGCCATGGAACAAAAACTAATATCAGAAGAAGACCTAGCTGATATGCGTAGACGAAATGGAAG-3’  5’-GAAGGCCTCTACATCATATCTCTGACACGTTTCG-3’  5’- CCCACCTGAGTTTGTGGACT-3’  5’-TTTTACCCACCCAAACCAAA-3’  5’-ACTTAATTAGCACTCAAATTAAACAAAATAAGT-3’  5’-TTTAAACATAAGAAGAAGTTCCTTTTTCATCTAC-3’  5’-GGTTTTATATTAATATTAAAGAGTTATGGGTYGAAGTTT-3’  5’-CAAAATACTTTACACATAAACRAAAAACAAACAAATCRAA-3’  5’-AACCAAAATCATTCTCTAAACAAAATATAAAAAAATC-3’ |
